# LinTT1-Functionalized Hybrid Lipid–Polymer Nanoparticles for Glioblastoma Targeting

**DOI:** 10.1021/acsptsci.5c00537

**Published:** 2025-09-30

**Authors:** Antonella Rocchi, Valeria Sidorenko, Nicola d’Avanzo, Luca Marchetti, Jhalak Sethi, Luigi Ciriolo, Anna Maria Tolomeo, Maria Grazia Cifone, Paola Palumbo, Massimo Fresta, Tambet Teesalu, Christian Celia

**Affiliations:** † Laboratory of Precision and Nanomedicine, Institute of Biomedicine and Translational Medicine, 533907 University of Tartu, Ravila 14b, Tartu 50411, Estonia; ‡ Department of Pharmacy, University of Chieti − Pescara “G. d’Annunzio”, Via dei Vestini 31, Chieti 66100, Italy; § Department of Clinical and Experimental Medicine, 199924 University of Catanzaro “Magna Græcia”, V.le “S. Venuta”, Catanzaro I-88100, Italy; ∥ Research Center “ProHealth Translational Hub”, Department of Experimental and Clinical Medicine, “Magna Graecia” University of Catanzaro, Campus Universitario “S. Venuta” − Building of BioSciences, Viale S. Venuta, Catanzaro I-88100, Italy; ⊥ Department of Health Science, University of Catanzaro “Magna Græcia”, V.le “S. Venuta”, Catanzaro I-88100, Italy; # Department of Cardiac, Thoracic and Vascular Science and Public Health, University of Padova, Padua I-35128, Italy; ∇ Perdiatric Research Institute ″Città della Speranza″, Corso Stati Uniti, 4, Padua I-35127, Italy; ○ Department of Life, Health & Environmental Sciences, 489868 University of L’Aquila, Via Pompeo Spennati, Building Rita Levi Montalcini, Coppito, L’Aquila I-67100, Italy; ◆ Materials Research Laboratory, University of California, Santa Barbara, California 93106, United States; ¶ Institute of Nanochemistry and Nanobiology, School of Environmental and Chemical Engineering, Shanghai University, Shanghai 200444, China; & SCM Nutraceutici Universitari Srl, Strada degli Oliveti 73, Chieti I-66100, Italy; ● Division of GI/Endocrine Surgery, Department of Surgery, Columbia University Irving Medical Center, New York, New York 10032, United States

**Keywords:** hybrid nanoparticles, nanomedicine, glioblastoma multiforme, CendR peptide, tumor homing peptides, precision medicine

## Abstract

Glioblastoma multiforme (GBM) is an aggressive brain tumor with limited therapeutic options and a poor prognosis. We developed hybrid lipid-polymer nanoparticles (HLPNs) functionalized with tumor-homing C-end Rule peptide LinTT1 (LinTT1-HLPNs) to improve the GBM targeting. *In vitro* studies demonstrated that LinTT1-HLPNs bind selectively to GBM cells and significantly improved the cytotoxicity of the loaded temozolomide (TMZ) (LinTT1-HLPNs@TMZ) compared to that of the free drug. *In vivo*, intravenous injection of HLPNs in both infiltrative and noninfiltrative GBM murine models had an enhanced accumulation of TMZ in the tumor area, thus endorsing the selective targeting and tissue penetration of LinTT1-HLPNs. This nanoplatform combines the advantages of hybrid lipid–polymer nanoparticles with a GBM-specific targeting strategy, thus providing an improved drug delivery and therapeutic effect by a multistep targeting approach, which addresses the key challenge of GBM.

## Graphical abstract

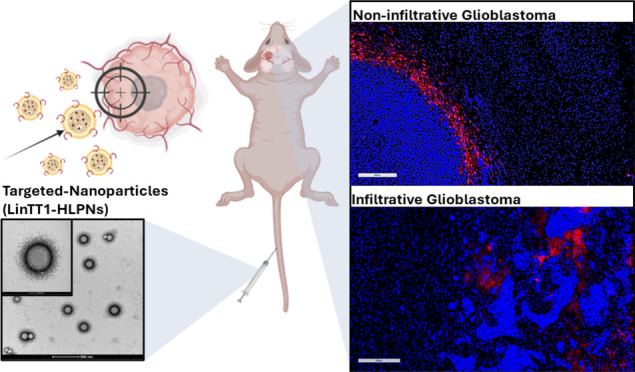

Glioblastoma multiforme (GBM) is the most aggressive and prevalent primary brain tumor in adults, and it is characterized by a rapid growth and a highly infiltrative nature.^1^ The prognosis for GBM patients is poor, with a median survival of 12 to 15 months postdiagnosis, a high tumor recurrence rate,^2^ and a five-year survival rate below 5%.^3^ The standard treatment for GBM has remained unchanged since 2005,^4^ including the surgical resection followed by radiation therapy and adjuvant temozolomide (TMZ) treatment.^5^ However, TMZ efficacy is limited by the DNA mismatch repair (MMR) system, leading to drug resistance in approximately 90% of patients.^5,6^ The induction of drug resistance, dose-limiting myelosuppressive toxicity, and fast *in vivo* inactivation^7^ represent significant challenges in GBM treatment.^8^ Precision-guided nanoparticles have been explored to improve the pharmacokinetic properties and therapeutic index of GBM drugs.^9,10^ Recent studies have harnessed peptide-functionalized PEGylated and targeted immunoliposomes to deliver drugs, small-molecule inhibitors or siRNA, achieving enhanced BBB penetration and significant increases in median survival in orthotopic GBM models.^11–13^ Parallel efforts with PLGA-based polymeric nanoparticles have enabled sustained release of chemotherapeutics,^14^ yielding marked GBM growth inhibition and reduced off-target toxicity.^15,16^ Based on the clinical success of polymeric nanoparticles and liposomes,^17^ hybrid lipid-polymer nanoparticles (HLPNs) have emerged as a promising drug delivery platform for GBM.^18^ HLPNs combine the structural and functional advantages of both material classes, offering high drug encapsulation efficiency, tunable and sustained drug release profiles, robust serum stability,^19^ and the capacity for surface functionalization to enable targeted delivery to specific cells and tissues.^20^

The C-end Rule (CendR) tumor-penetrating peptides, such as the cryptic iRGD (CRGDKGPDC),^21^ exhibit tumor specificity through their reliance on tumor-enriched molecular features like proteases and receptors.^22^ In the case of iRGD, this process begins with the peptide homing to angiogenic integrins expressed on the tumor vasculature (e.g., αvβ3/β5).^23^ The peptide is then proteolytically cleaved to expose a cryptic CendR motif (R/KXXR/K), which enables subsequent binding to neuropilin-1/2 (NRP-1/2)^24^ and triggers tissue penetration of both the peptide and its conjugated or coadministered payload.^25,26^ iRGD has been widely used to enhance tumor delivery of diverse anticancer agents, including small-molecule drugs, monoclonal antibodies, and a variety of nanoparticle formulations, and is undergoing clinical trials for multiple solid tumor types worldwide.^27–30^

LinTT1 (Linear TT1; AKRGARSTA) is linear derivative of cyclic TT1 peptide (CKRGARSTC) that was identified by cell-free peptide phage biopanning on recombinant p32,^31^ a mitochondrial protein expressed on the surface of activated cells in tumors such as macrophages, endothelial cells, and malignant cells in several solid tumors, including in GBM.^32–35^ As with iRGD and other tumor-penetrating peptides, LinTT1 follows a multistep pathway to accumulate in and penetrate tumors. After LinTT1 binding to its cell-surface receptor p32, a serine protease overexpressed in many solid tumors, urokinase-type plasminogen activator (uPA)^36^ cleaves the peptide to C-terminally expose the CendR motif R/KXXR/K–OH (AKRGAR in LinTT1).^37^ The CendR motif then engages with NRP-1/2 to trigger transcytosis, extravasation, and deep penetration of peptide together with the cargo into the tumor parenchyma.^38–41^

Our current study explored the applicability of LinTT1-guided HLPNs for precision targeting of GBM. We developed LinTT1-functionalized HLPNs and evaluated them both *in vitro* and *in vivo*, demonstrating effective multistep targeting that enhanced brain penetration and enabled delivery of the particles to deep regions of the GBM parenchyma.

## 1 Results and Discussion

### 1.1 Physicochemical Properties

Physicochemical properties of nanoparticles strongly affect their *in vivo* circulation, accumulation, and metabolism.^42^ After conjugation of FAM (6-carboxyfluorescein)-labeled LinTT1 peptides to HLPNs, the (LinTT1-HLPNs) average size of particles increased from 155.5 to 161.6 nm and the zeta potential decreased from −32.6 to −27.3 mV (Figure 1A). These changes are consistent with successful LinTT1 peptide conjugation, which increased the water layer around the HLPNs and reduced their surface negative charge due to the peptide’s positive charge (Figure 1A). The conjugation efficiency of LinTT1 with HLPNs was 89.9%, as quantified by fluorimetry (Figure 1B). As indicated by the polydispersity index (PDI) values of HLPNs and LinTT1-HLPNs, which were both below 0.1, the nanoparticles had a narrow size distribution, which is a property of uniform and monodisperse nanoparticles^43^ (Figure 1A). Dynamic light scattering (DLS) data was confirmed and complemented by transmission electron microscopy (TEM) analysis, which also verified the spherical shape of both HLPNs and LinTT1-HLPNs (Figure 1C). Furthermore, TMZ-loaded HLPNs, HPLNs@TMZ, and LinTT1–HLPNs@TMZ had shapes and physicochemical properties similar to those of empty HLPNs and LinTT1–HLPNs (Table S1). The net negative zeta potential for LinTT1-HLPNs@TMZ indicates that HPLNs maintain colloidal stability over time,^44^ in agreement with minimal size variation observed during 4 weeks of incubation at 4 °C (Figure 1D). To further assess the stability of LinTT1-HLPNs@TMZ under conditions mimicking the *in vivo* microenvironment, the HPLNs were incubated in 50% (v/v) human plasma and the size was monitored over 72 h. We showed that LinTT1-HPLNs@TMZ incubated with PBS were stable for up to 72 h, maintaining an average size of ∼168 nm. In contrast, LinTT1-HLPNs@TMZ, incubated in human plasma showed time-dependent size changes (Figure 1E). They remained stable for the first 8 h, but their average size increased significantly thereafter, more than twice after 24 h (Figure 1E). Consistently, the polydispersity index (PDI) increased significantly after 8 h, from ∼0.10 to >0.40, thus showing a reduced narrow size distribution. In parallel, the zeta-potential was stable at −27 mV up to 8 h but had less negative values, as −11.9 mV, at 24 h, and −9.4 mV at 72 h (Table S2). This effect is likely to depend on the formation of hard and soft protein coronas on the surface of nanoparticles^45,46^ and their supramolecular architecture, with a rigid and internal PLGA polymer core and a PEGylated lipid monolayer at the interface, that effectively prevents nanoparticle aggregation.^47^ In particular, the PEG coating acts as a stabilizing layer that minimizes the protein adsorption, maintaining the nanoparticle’s stability in biological environments during the early incubation, prior to hard corona formation by 24 h of incubation.^48^ These results demonstrate that LinTT1-HLPNs@TMZ remains stable in human plasma after 8 h of incubation, a time frame sufficient for targeted delivery after the intravenous administration. This combination of lipid and polymer components confers overall stability,^49^ making these nanoparticles a promising candidate for systemic GBM therapy.^50^ The comparative studies showed that the HLPNs have higher colloidal stability and controlled drug release than bare lipidic or polymeric nanoparticles, and the relative increase of nanoparticle stability depended on the synergistic properties of biomaterials.^51–53^

**Figure 1:**
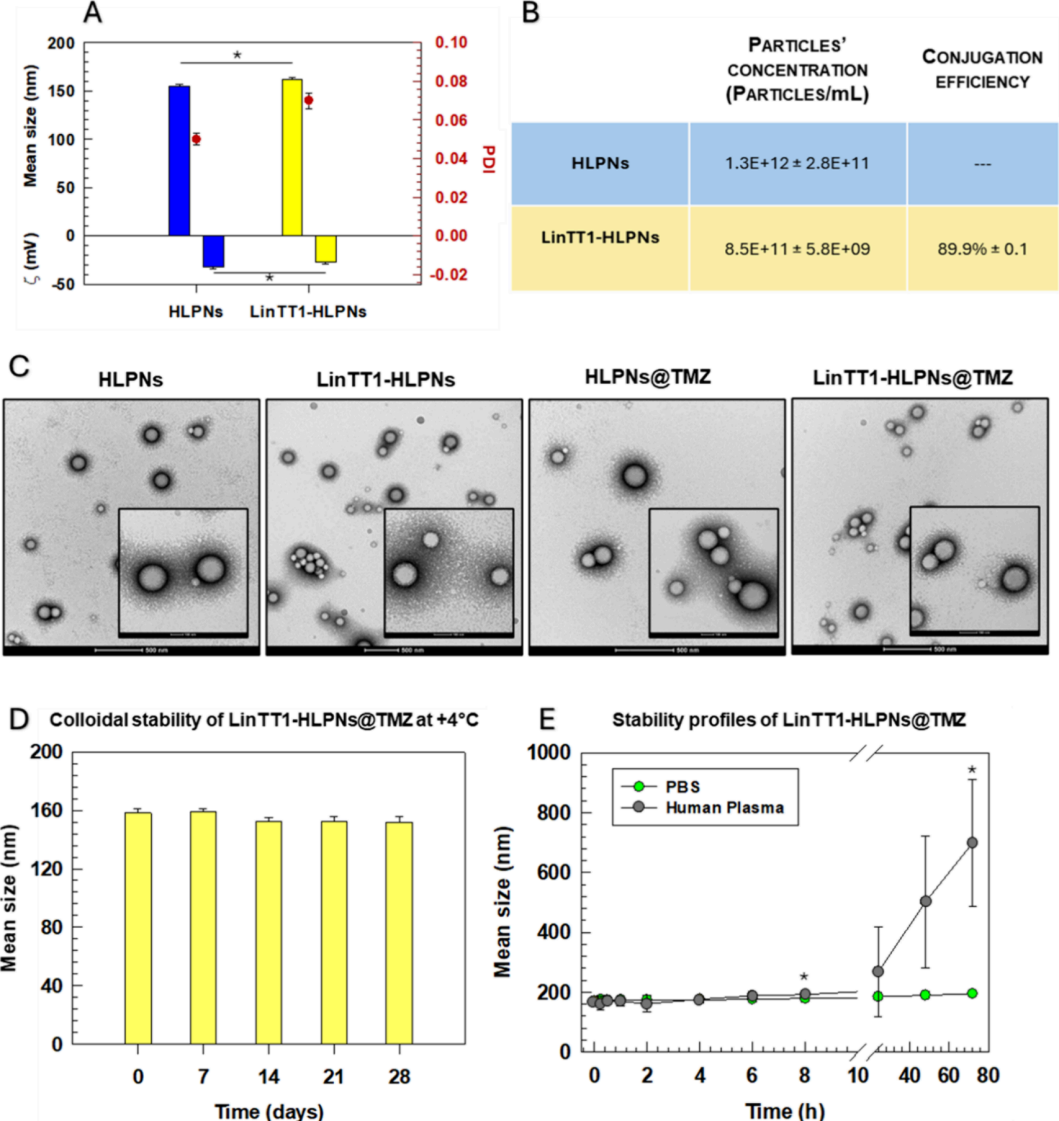
Physicochemical properties of different HPLN formulations. (A) Average hydrodynamic diameter, zeta potential, and polydispersity index (PDI) of HLPNs before and after surface conjugation with the LinTT1 peptide. (B) Particle concentration (particles/mL) and LinTT1 conjugation efficiency. (C) Representative TEM images of HLPNs and LinTT1-HLPNs with and without TMZ at different magnifications (scale bar: 500 and 100 nm). (D) Colloidal stability of LinTT1-HLPNs@TMZ after storage in PBS (pH 7.4) at 4 °C for up to 4 weeks. (E) Stability profiles of LinTT1-HLPNs@TMZ incubated in PBS (pH 7.4) and human plasma (50% v/v) at 37 °C. Results are the mean of three independent experiments ± standard deviation (S.D.) *p < 0.05. **p < 0.01. ***p < 0.001 [one-way analysis of variance (ANOVA)].

Similar data was obtained with nonfunctionalized FAM-HLPNs@TMZ, confirming that LinTT1 functionalization does not compromise nanoparticle stability (Figure S1A,B).

### 1.2 Encapsulation Efficiency and Release Kinetics

To evaluate the drug-carrying performance of nontargeted HLPNs, we assessed their entrapment efficiency and investigated the release kinetics of TMZ under physiologically relevant conditions. The entrapment efficiency of TMZ within the HPLNs was 35 ± 1.8%, with a drug loading of 5.93 ± 0.2%, when a solution of TMZ at a concentration of 4 mg/mL was used during the preparation procedure (Figure 2B). LinTT1-HPLNs@TMZ had similar values of entrapment efficiency and drug loading, i.e., 33.9 ± 0.7 and 5.74 ± 0.1, and demonstrated that the conjugation of LinTT1 on the surface of HPLNs does not modify the entrapment efficiency and the loading of nanoparticles. These results are consistent with previous data on TMZ encapsulation in polymeric and hybrid nanoparticles and therefore these particles were selected for further experiments.^54^

**Figure 2:**
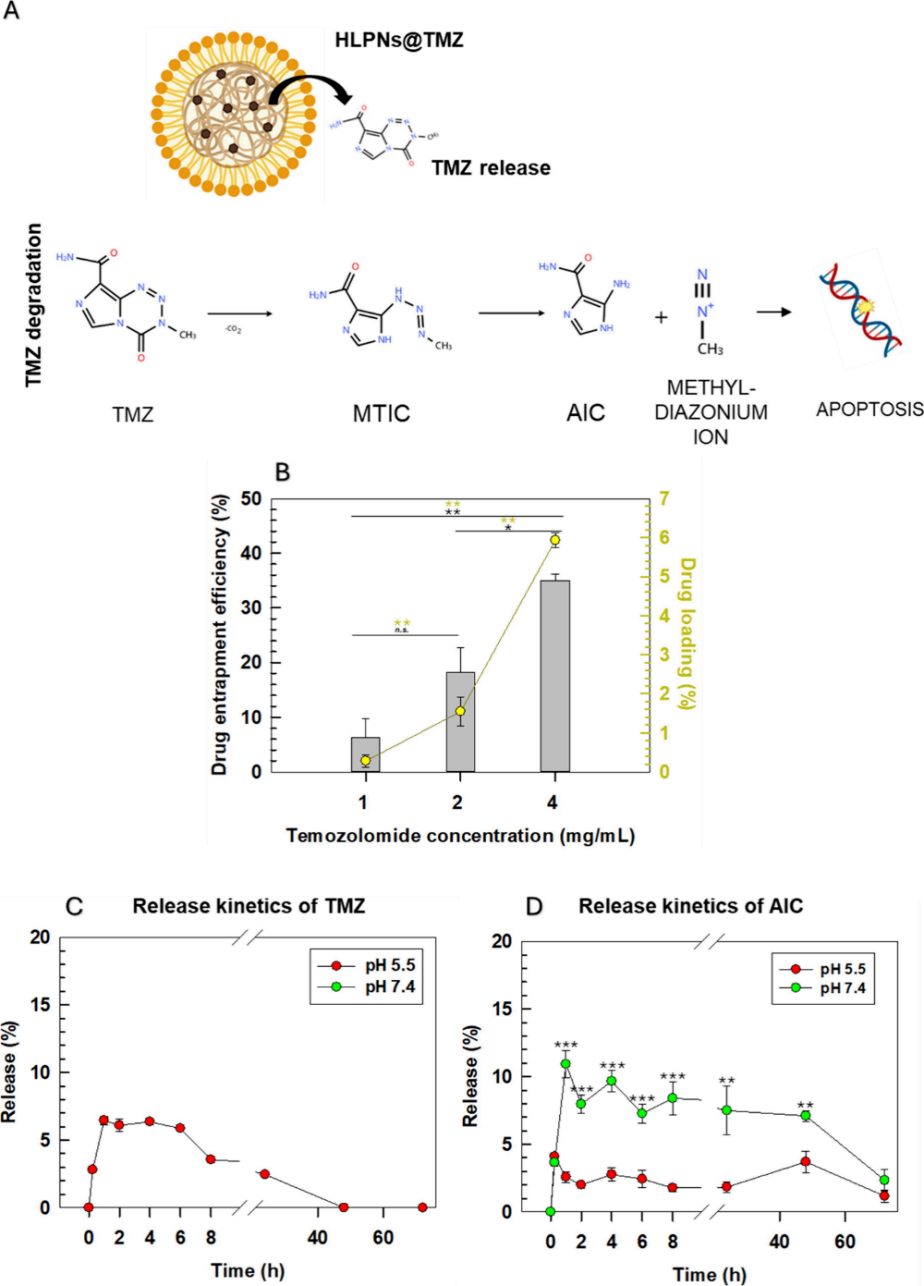
Encapsulation efficiency and release kinetics of nontargeted HPLNs@TMZ: (A) Schematic description of TMZ hydrolysis under physiological pH (7.4), yielding 5-(3-methyltriazen-1-yl)­imidazole-4-carboxamide (MTIC), 5-aminoimidazole-4-carboxamide (AIC), and methyl diazonium ion. (B) Drug entrapment and loading efficiency were determined by varying the TMZ concentration during the preparation procedure of HLPNs. (C) *In vitro* release profile of TMZ at pH 5.5 and 7.4. Under physiological conditions, TMZ was not detectable in its prodrug form due to rapid hydrolysis, and only its metabolite AIC was quantified. (D) Release kinetics of the AIC (TMZ metabolite) at pH 5.5 and 7.4. Results are the mean of three independent experiments ± standard deviation (S.D.) *p < 0.05. **p < 0.01. ***p < 0.001 [one-way analysis of variance (ANOVA)].

Clinically, the standard Stupp protocol^4^ for TMZ recommend the using of 75 mg × m^–2^/day during radiotherapy followed by 150–200 mg × m^–2^/day for 5 days every 28 days. Since the drug loading of HLPNs is 6% w/w, we tested the range of concentration for TMZ as reported in published liposomes^55^ and polymeric nanoparticles.^56^ Noteworthily, HLPNs have some translational advantages compared to free TMZ: (i) enhanced drug protection from premature degradation, (ii) sustained release kinetics, and (iii) selective delivery to GBM cells by targeting peptide-receptors. These properties are expected to significantly improve the effectiveness of TMZ by decreasing, at the same time, the systemic side-effects and thus potentially injecting a lower dosage of drug.^57^

The release kinetics of TMZ from HPLNs was evaluated in PBS at two different pH values, 7.4 and 5.5, to mimic the physiological and acidic tumor microenvironments, respectively (Figure 2C,D). Since LinTT1 functionalization is obtained after conjugation of the peptide on the external surface of TMZ@HLPNs, the internal structure and release properties of HLPNs are not significantly affected; therefore, the release profile of bare-HLPNs (untargeted HLPNs) is representative of LinTT1-HLPNs.

The release of TMZ and its metabolite 5-aminoimidazole-4-carboxamide (AIC) was monitored over time to evaluate the potential degradation of TMZ at pH 7.4 and 5.5 and its conversion to the relative drug metabolite^58^ (Figure 2A). The release of TMZ was negligible at pH 7.4, likely due to its instant conversion into the AIC metabolite. The rapid degradation of TMZ, in physiological conditions, is consistent with its instability in aqueous buffer at neutral pH, and therefore the drug release at pH 7.4 was monitored indirectly by quantifying its metabolite AIC.^59^ At pH 5.5, ∼7% TMZ was released within the first hour followed by pseudo-steady state release kinetics up to 6 h. After 8 h of incubation, the TMZ concentration in the receptor medium gradually decreased due to its degradation (Figure 2C). In agreement with these results, the release kinetics of AIC at pH 7.4 was significantly higher than that observed at pH 5.5 (Figure 2D). This is probably due to the degradation of TMZ at pH 7.4 and its conversion to AIC.^60^ At both pH levels, the concentration of AIC remained stable for up to 48 h; however, a significant decrease was observed at 72 h, likely due to oxidative reaction.^61^ Since LinTT1 functionalization is performed after drug loading on the nanoparticle exterior, the release profile of bare-HLPNs is considered representative of LinTT1-HLPNs.

At early incubation times (≤8 h), the cumulative amount of TMZ and AIC measured at pH 5.5 was like the total amount of AIC quantified at pH 7.4, where TMZ is quickly degraded (Figure 2C,D). At pH 5.5, TMZ is widely stable, and only a minor fraction is converted into AIC, whereas at pH 7.4 the TMZ is degraded (hydrolysis) after a few hours of incubation. This result shows that the overall fraction of the drug released from HLPNs is pH-independent and corresponds to ∼10% of the encapsulated payload, while the detectable compound (intact TMZ vs AIC) represents the distinct chemical stability of TMZ under neutral and acidic conditions. Although this relatively slow release may be suboptimal for drug delivery, it can provide clinical advantages. Like for other approved nanomedicines, such as Caelyx,^62^ a slow-release profile can reduce the premature drug leakage during blood circulation while still enabling effective release at the target site in response to enzymatic or physicochemical stimuli.^63^

The mathematical modeling of the release kinetics was carried out to study the mechanistic release properties of temozolomide (TMZ) and its metabolite AIC under different experimental conditions (Figure S2). For TMZ, kinetic fitting was restricted to the early time window (0–6 h), as this interval represents the linear or quasi-linear portion of the release profile where classical models (zero-order, first-order, Higuchi, Korsmeyer–Peppas) are valid descriptors of controlled release processes. In this range, the first-order model provided the best fit, supporting a diffusion release mechanism through the polymeric-lipid membrane of HLPNs (Figure S2A and Table S3). Conversely, at longer incubation time points (>8 h), the conventional models failed to describe the overall release trend due to TMZ degradation in the release medium. In these attempts, the nonmonotonic release is better described by burst-decay model. This last approach highlighted both the initial release and the subsequent loss of active drug due to chemical instability of TMZ (Figure S2B and Table S3). For AIC, the release at physiological pH displayed sufficient linearity to be analyzed within the 0–6 h interval, where a first-order model yielded the most appropriate description, even if with the fitting remained moderate (Figure S2C and Table S3). In contrast, at pH 5.5, the release profile was highly irregular, characterized by an initial burst followed by fluctuating values without a clear monotonic trend. This prevented the application of conventional kinetic models

### 1.3 Binding and Activation Properties of LinTT1 in a Cell-free System and *In Vitro*

Peptide conjugation to nanoparticles can potentially hinder the peptide’s ability to engage with its target receptor, thereby reducing target efficiency. To ensure that LinTT1 retains its targeting functionality after conjugation to HLPNs, we evaluated its ability to engage with its receptors in a cell-free system. We examined the selective binding of LinTT1-HLPNs to the p32 receptor followed by the exposure of the cryptic CendR motif required for NRP-1 engagement (Figure 3A). Magnetic beads were coated with the recombinant p32 and b1b2 domain of NRP-1. LinTT1-HPLNs (FAM-LinTT1-HPLNs) and nontargeted HPLNs (FAM-HLPNs) were incubated with immobilized receptors and then quantified by measuring the fluorescence. To mimic the *in vivo* multistep pathway of LinTT1, uPA was included in the assay to cleave LinTT1 and expose its cryptic CendR motif. The results demonstrate that LinTT1-HLPNs effectively bind to the p32 protein without uPA treatment (Figure 3B), while the uPA-mediated peptide cleavage exposes the CendR motif, enabling selective binding to NRP-1 ^64^ (Figure 3C). These results are in line with the binding of LinTT1-HLPNs with p32 and NRP-1 receptors following a key-to-lock interaction that depends on the position of amino acid motifs within the peptide.^65^ Notably, the binding of LinTT1-HLPNs to the NRP-1 domain is increased by uPA treatment approximately 4-fold.

**Figure 3:**
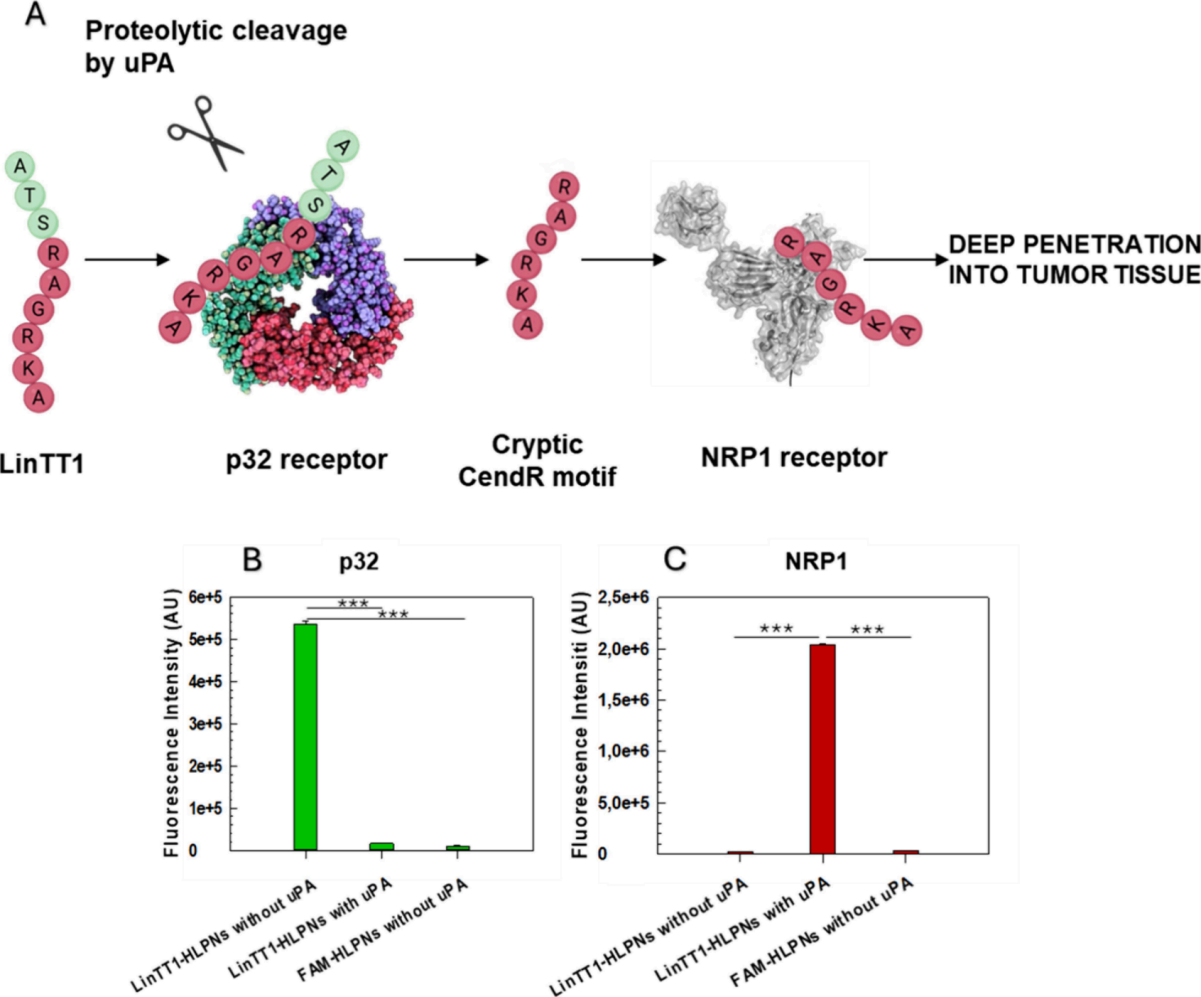
Binding and activation properties of LinTT1 in a cell-free system and *in vitro*: (A) Schematic description of the multistep tumor penetration mechanism mediated by the LinTT1 peptide. (B, C) Assessment of binding interactions between LinTT1-HLPNs and the p32 receptor and between the proteolytically exposed CendR motif and LinTT1-HLPNs and NRP1 following uPA-mediated cleavage. Results are the mean of three independent experiments ± standard deviation (S.D.) *p < 0.05. **p < 0.01. ***p < 0.001 [one-way analysis of variance (ANOVA)].

To assess whether peptide conjugation enhances nanoparticle uptake in cultured cancer cells, we first studied HLPNs functionalized with the prototypic CendR peptide,^66^ RPARPAR (FAM-cys-RPARPAR; in short RPAR), which has physicochemical properties similar to the LinTT1-HLPNs (Table S4). At this stage, RPAR was selected as a simplified model ligand that directly binds NRP-1, thus allowing the validation of nanoparticle internalization under controlled conditions before testing the more complex two-step mechanism of LinTT1. Like the uPA-cleaved LinTT1 peptide with an exposed and activated CendR motif (AKRGAR), RPAR has an active CendR motif that binds NRP-1/2 and hence has been widely used as a model NRP-1-targeting ligand.^67^ As a control, we used a scrambled variant of RPAR (scrRPAR, sequence RRAAPRP), which lacks the C-terminal CendR element and is therefore unable to bind the NRP-1. The binding and intracellular uptake of the RPAR-HLPNs was assessed in human prostate cancer cells (PPC-1)^68^ and human melanoma cells (M21),^69^ which overexpress and do not express the NRP-1 receptor, respectively.^70^ After incubation with peptide-targeted nanoformulations and washes to remove nonbound nanoparticles, only the PPC-1 cells had a strong fluorescent signal, in line with NRP-1-dependent cell targeting (Figure S3A). In contrast, scrRPAR-HLPNs were not significantly taken up by PPC-1 cells, in agreement with the requirement for CendR motif for NRP-1 receptor binding.^71,72^ No significant uptake of RPAR-HLPNs and scrRPAR-HLPNs was observed after 1 h of incubation with M21 cells (Figure S3A). Flow cytometry confirmed these findings, showing that approximately 90% of PPC-1 cells treated with RPAR-HLPNs were FAM-positive, while M21 cells showed minimal uptake (Figure S3B).

To further study the selectivity of the RPAR-HLPN interaction with the NRP-1 receptor, PPC-1 cells were preincubated with a panel of anti-NRP-1 monoclonal antibodies^70^ (Figure S3C,D). These antibodies included a blocking antibody (mAb7E8) capable of inhibiting the CendR binding site and a nonblocking antibody (mAb3E7).^73^ When PPC-1 cells were preincubated with the blocking antibody, the uptake of RPAR-HLPNs was significantly decreased at both tested antibody concentrations (0.01 and 0.02 mg/mL). In contrast, when preincubated with the nonblocking antibody, the effect on RPAR-HLPN binding and uptake was minimal (Figure S3C,D).

These results further support the selective uptake of RPAR-HLPNPs by tumor cells through NRP-1-mediated endocytosis^74^ and suggest a role for the uPA-activated CendR motif of the LinTT1 peptide following uPA processing.

The first step of LinTT1-HLPN interaction with cancer cells and the tumor microenvironment is mediated by the p32 protein, which is expressed on the cell surface under metabolic stress.^33^ We studied the p32-mediated uptake of LinTT1-HLPNs by wtGBM, VEGFko-GBM, and GL261 cells under normal conditions and nutrient starvation (Figure 4A–C). Compared to standard cell culture conditions, serum starvation induced a 3-fold increase in p32 surface expression in both VEGFko-GBM and wtGBM cells (Figure 4A,B), which was characterized by a marked rise in LinTT1-HLPN uptake from 4.9% ± 0.2% to 60% ± 1.2% in VEGFko-GBM cells (Figure 4D), and from 7.5% ± 0.8% to 60.0% ± 2.8% in wtGBM cells (Figure 4E). A slight increase in the uptake of FAM-HLPNs was observed under starvation conditions (Figure 4D,E).

**Figure 4:**
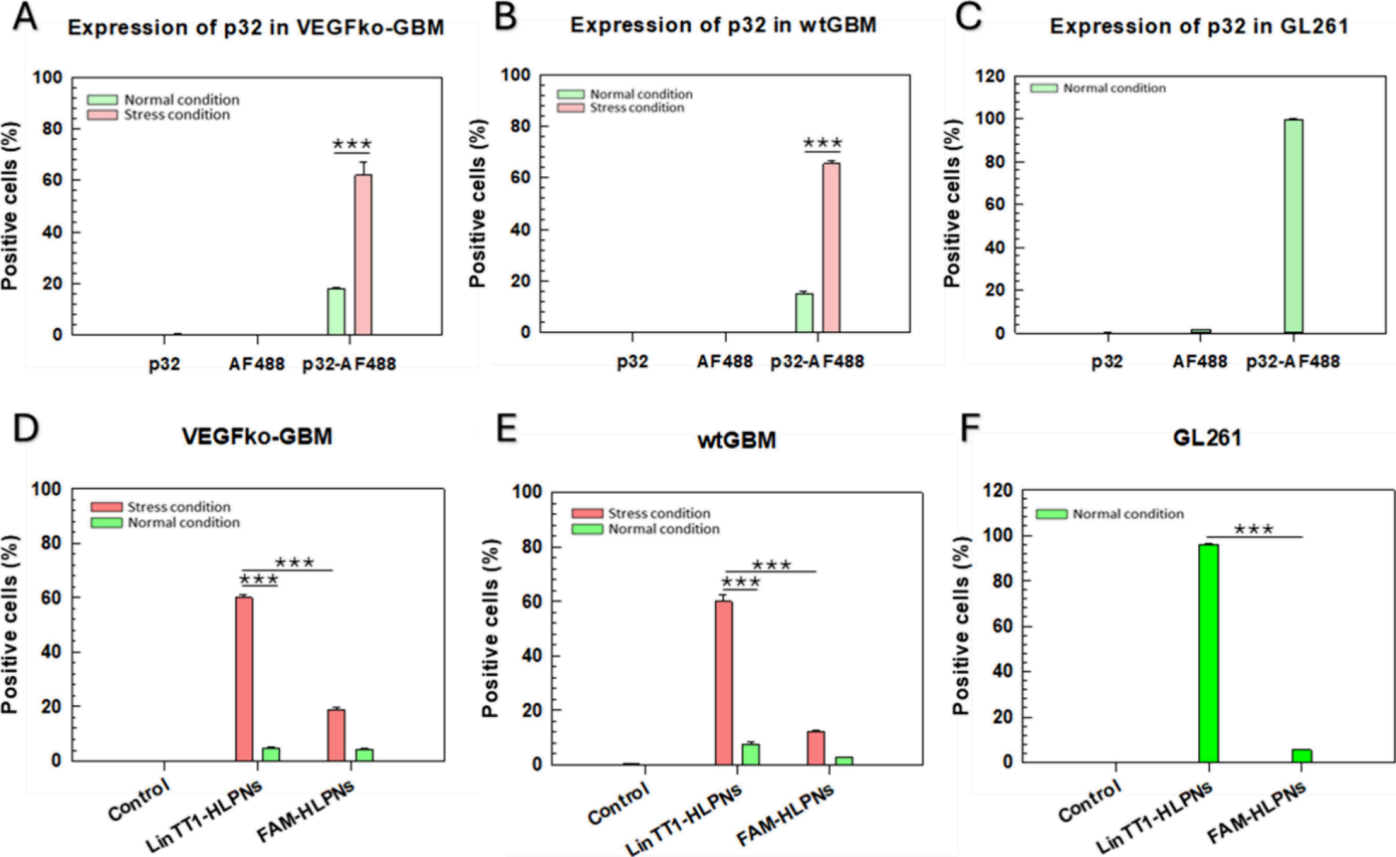
Expression of p32 receptor *in vitro* in GBM cells and *in vitro* binding. (A–C) Flow cytometry analysis of the p32 receptor surface in GBM cell lines under normal and nutrient-deprivation conditions. Cells were stained with a p32-specific primary antibody (p32) and a fluorescently labeled secondary antibody (Alexa Fluor 488, AF488), termed p32-AF488 in the graphs. Controls including cells stained with the p32 antibody alone or AF488 secondary antibody showed no detectable fluorescence, confirming that the signal in p32-AF488 corresponds to the specific labeling of p32. (A) VEGFko-GBM, (B) wtGBM, and (C) GL261 lines. Data represent mean ± standard deviation (S.D.) from three independent experiments (*n* = 3). (D–F) *In vitro* binding evaluation of LinTT1-HLPNs and FAM-HLPNs under normal and stress (oxidative) conditions in (D) VEGFko-GBM, (E) wtGBM, and (F) GL261 cell lines. Results are the mean of three independent experiments ± standard deviation (S.D.) *p < 0.05. **p < 0.01. ***p < 0.001 [one-way analysis of variance (ANOVA)].

In contrast, GL261 cells that show high *in vitro* expression of the p32 protein in normal conditions, with 99.7% ± 0.2% of cells being of p32-positive (Figure 4C), take up preferentially LinTT1-HLPNs (96.03% ± 0.17% cells positive) over FAM-HLPNs (<5% cells positive) (Figure 4F).

These results confirm that cell surface expression of p32 in wtGBM and VEGFko-GBM is associated with nutrient-deprived environments and that LinTT1-HLPN uptake is correlated with the presence of this receptor on the cell surface.^34,75^

### 1.4 *In Vitro* Cytotoxic Effects

To evaluate the therapeutic potential of LinTT1-targeted nanoparticles following receptor-mediated uptake, we next assessed their cytotoxic effects *in vitro*. The effect of LinTT1-HLPNs@TMZ, FAM-HLPNs@TMZ and free TMZ on viability of GBM cell lines was tested across the concentration range of 2.5 to 50 μM (Figure 5A–C) based on the TMZ encapsulated in the nanoparticles according to the resulting entrapment efficiency of drug and represents the total encapsulated drug rather than the fraction released during the assay. After 1 h incubation, GBM cells were washed after which fresh medium was added for an additional 72 h incubation. This setup was intended to mimic *in vivo* conditions, where factors such as sink effects, enzymatic degradation, and physiological clearance limit prolonged nanoparticle–tissue interactions.^76^ As shown in Figure 5, free TMZ did not have significant cytotoxic effects on GBM cells at any tested concentration. This lack of activity is likely due to the short half-life of the drug and its rapid conversion to the active form (AIC) at physiological pH, which limits its efficacy,^77^ and the short incubation time between cells and TMZ (Figure 5A–C).

**Figure 5:**
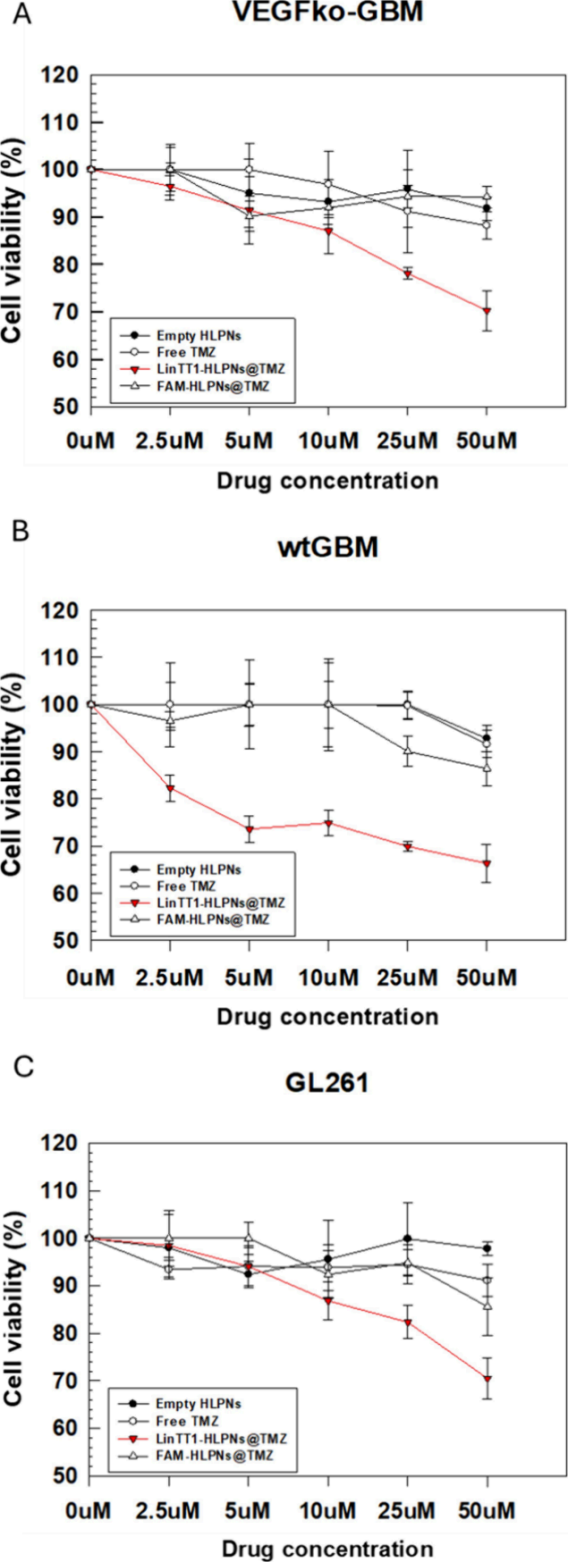
*In vitro* cytotoxic effects of empty HLPNs, free TMZ, LinTT1-HLPNs@TMZ, and FAM-HLPNs@TMZ in (A) VEGFko-GBM, (B) wtGBM, and (C) GL261 cells treated with TMZ in the range of concentrations from 2.5 to 50 μM. Results are the mean of three independent experiments ± standard deviation (S.D.). Further statistical data analyses are available in the Supporting Information (Table S3).

In contrast, LinTT1-HLPNs@TMZ exhibited a dose-dependent cytotoxicity in all three GBM cell lines (Figure 5A–C). LinTT1-HLPNs@TMZ protects the TMZ prodrug until cellular uptake, where TMZ is converted to AIC. Cellular viability assays demonstrated significant reduction following LinTT1-HLPNs@TMZ treatment and a negligible effect following treatment with FAM-HLPNs@TMZ (Figure 5A–C). In particular, LinTT1-HLPNs@TMZ at 5 μM was more toxic for wtGBM cells than VEGFko-GBM cells, with a survival rate of 73.6% in wtGBM cells and 91.4% in VEGFko-GBM cells (Figure 5A,B). This trend was observed for TMZ concentrations of up to 50 μM, with a survival rate of 66.3% (wtGBM) and 70.3% (VEGFko-GBM) (Figure 5A,B). The cell lines used in this study give rise to malignant astrocytomas *in vivo*, with VEGF knockout (VEGFko) tumors reported to grow more slowly than wtGBM tumors in the brain parenchyma, resulting in prolonged mean survival in mice.^78^ VEGF is a key regulator of angiogenesis^79,80^ and plays a crucial role in cancer cell proliferation *in vitro*.^81,82^ Our observation on the increased sensitivity of wtGBM cells to LinTT1-HLPNs@TMZ suggests that this may be particularly suited for the treatment of aggressive, angiogenic GBM variants.

Interestingly, GL261 cells, despite displaying high expression of the p32 receptor (Figure 4C), exhibited only moderate sensitivity to LinTT1-HLPNs@TMZ. This may be attributed to their relatively slow proliferation rate, which could limit the cytotoxic efficacy of TMZ, which primarily targets dividing cells. LinTT1-HLPNs@TMZ showed a concentration-dependent cytotoxic effect on GL261 for the full drug range of concentration, with the highest efficacy at 50 μM. However, at the highest concentration of drug, no significant differences were obtained between targeted and untargeted HPLNs, maybe due to the static experimental conditions, which hindered the differences depending on the targeting properties of linTT1-HLPNs. Conversely, LinTT1-HLPNs@TMZ showed significant higher cytotoxic effect compared to FAM-HLPNs@TMZ at 25 μM, thus suggesting that in a monolayer GL261 cell *in vitro* model, this is the highest concentration, which allowed the greatest efficacy without any cytotoxic effect depending of the empty nanoparticles. At 50 μM, although the absolute cell viability was lower, the difference between LinTT1-HLPNs@TMZ and FAM-HLPNs@TMZ did not reach statistical significance (Table S5), likely due to reduced selectivity at higher drug concentrations (Figure 5C).

### 1.5 *In Vivo* Binding

Systemically administered CendR peptide-functionalized nanoparticles are known to exhibit lung tropism, making them a useful platform for initial proof-of-concept studies aimed at validating receptor-mediated accumulation of novel nanoplatforms.^83,64^ Therefore, a pilot *in vivo* study was performed in healthy mice using RPAR-HLPNs, a prototypical CendR-targeting system, to assess their pulmonary distribution prior to moving on to the more complex tumor-targeting properties of LinTT1-HLPNs. Consistent with the expected expression of neuropilin-1 in lung tissue, RPAR-HLPNs showed increased pulmonary accumulation compared to scrRPAR-HLPNs (Figures S4A,B). In addition, hepatic accumulation was observed, in line with normal hepatic clearance and metabolism of nanoparticles (Figures S5A,B). This data endorse the applicability of the peptide-guided nanoparticles for systemic targeting and provide the rationale for subsequent tumor-targeted studies with LinTT1.

Encouraged by these findings, we next evaluated the tumor-homing performance of LinTT1-HLPNs in orthotopic GBM xenograft models. The *in vivo* homing study on GBM xenograft models showed overall selective accumulation of LinTT1-HLPNs within the tumor area after intravenous administration (Figures 6 and 7). In the wtGBM model, characterized by localized growth of the tumor mass (Figure 6A), fluorescently labeled LinTT1-HLPNs were accumulating at the tumor border (Figure 6C). In addition, LinTT1-HLPNs were further detected in the deeper tumor area, particularly near the CD31-positive blood vessels (Figure 6D). The accumulation of LinTT1-HLPNs in the deeper tumor area correlated with overexpression of p32 in the hypoxic regions cells^34^ as well as by the cellular hitchhiking process as reported in a previous work.^84,85^ In fact, we have previously showed that LinTT1-targeted nanoparticles target M2-macrophages, the most abundant tumor-associated macrophages in solid tumors.^86,87^ The published data demonstrated targeted M2-macrophages that accumulated LinTT1-HLPNs in the deeper tumor layers by their shuttling properties and accumulation in the hypoxic area.^88^

**Figure 6:**
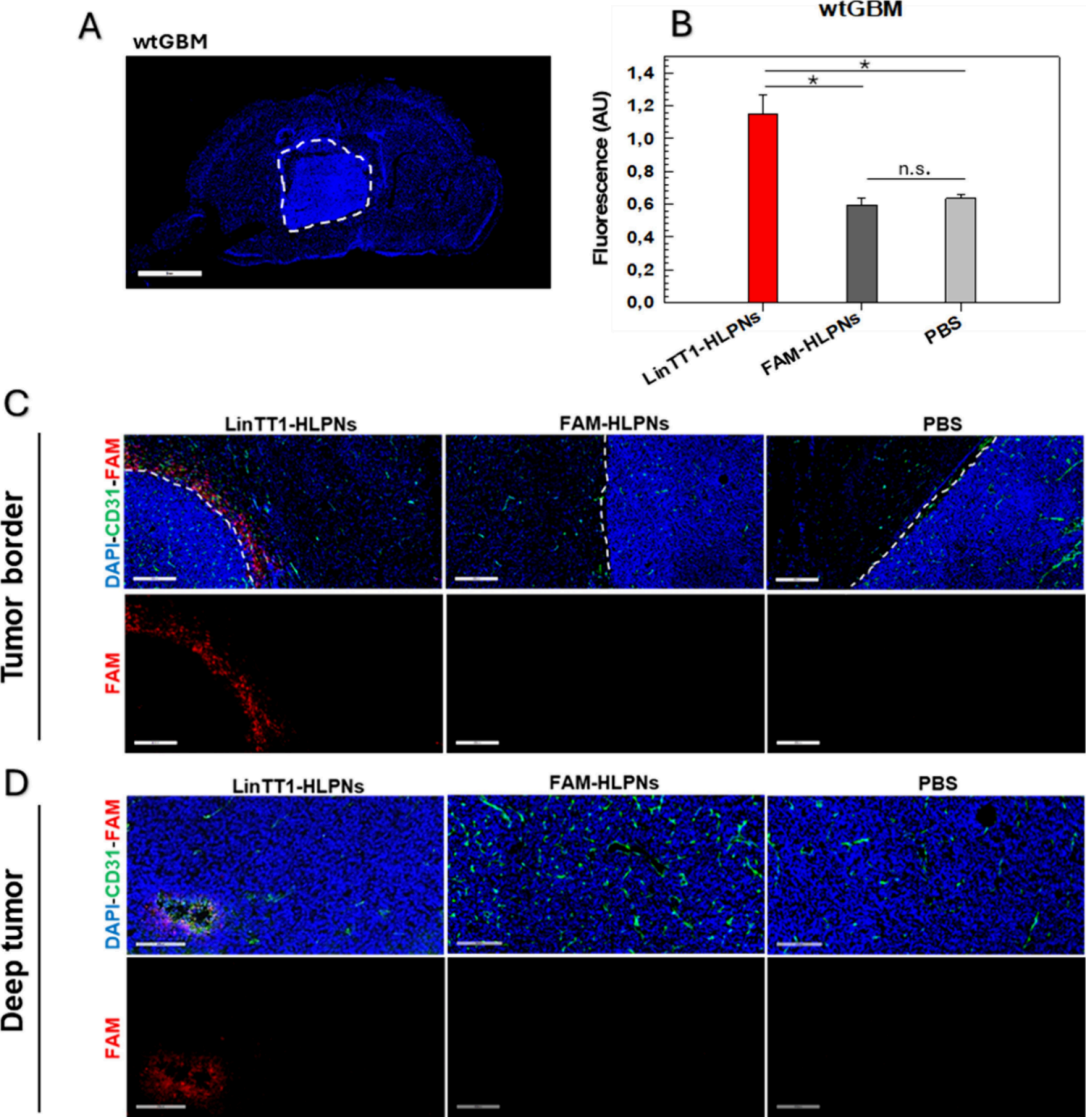
Confocal fluorescence imaging of (A) wtGBM (scale bar: 3 mm). (B) Quantification of fluorescence intensity in wtGBM was obtained from image analysis. (C, D) Tissue distribution after systemic administration of LinTT1-HLPNs and FAM-HLPNs in wild-type GBM (wtGBM) tumor-bearing mice, assessed at the (C) tumor border (scale bar: 400 um) and (D) in the tumor core (scale bar: 200 um). Tissues were collected 3 h postinjection, sectioned, and immune-stained for FAM (red, LinTT-HLPNs or FAM-HLPNs), CD31 (green, blood vessels), and DAPI (blue, nuclei). For each group, six tissue sections were analyzed per mouse (*n* = 6 sections) with three biological replicates (*n* = 3 mice per group). *p < 0.05. **p < 0.01. ***p < 0.001 [one-way analysis of variance (ANOVA)].

**Figure 7:**
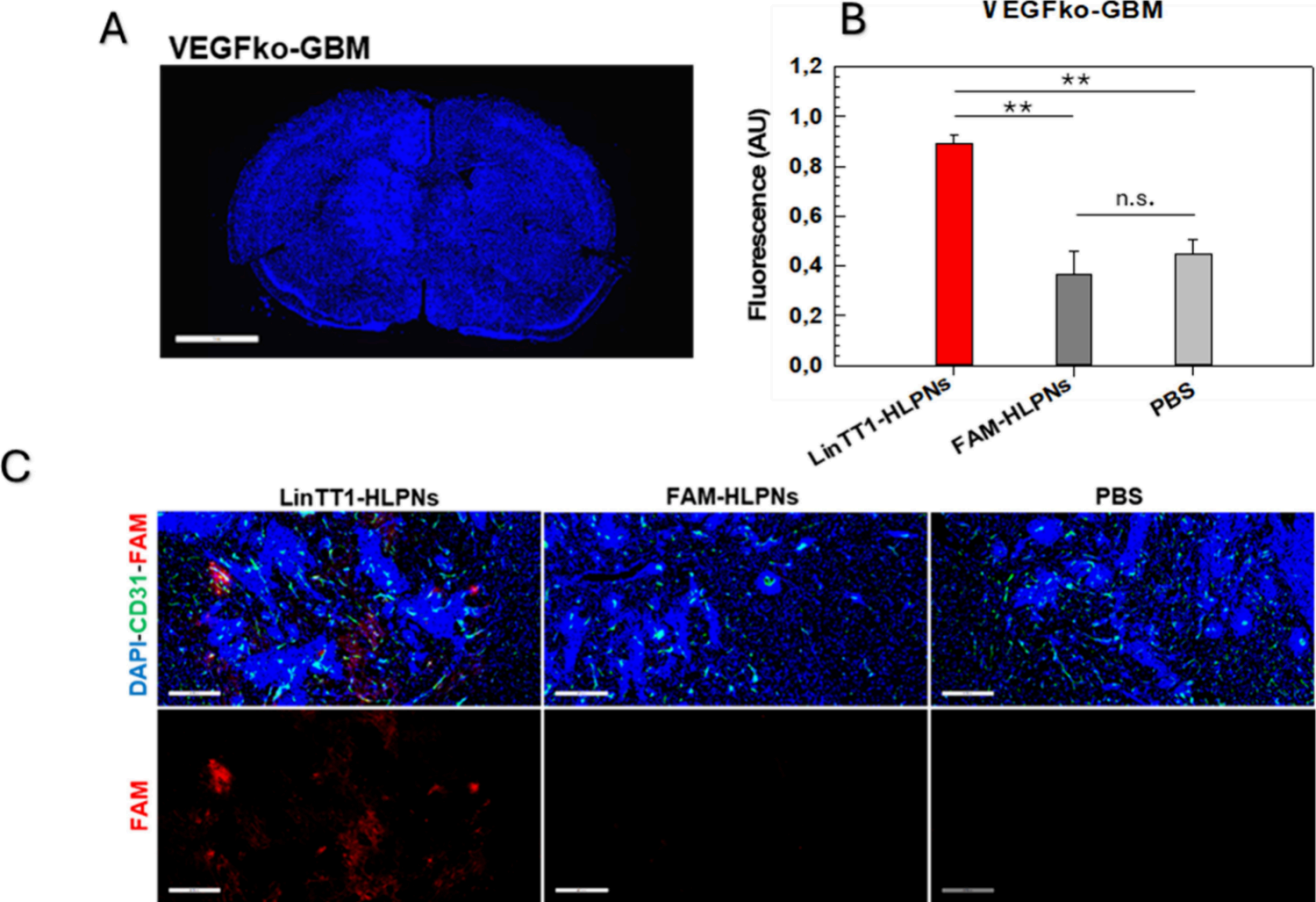
Confocal fluorescence imaging of (A) VEGFko-GBM (scale bar: 3 mm) tissues. (B) Quantification of fluorescence intensity in VEGFko-GBM obtained from image analysis. (C) Tissue distribution after systemic administration of LinTT1-HLPNs and FAM-HLPNs in VEGFko-GBM tumor-bearing mice (scale bar: 400 um). Tissues were collected 3 h postinjection, sectioned, and immune-stained for FAM (red, LinTT-HLPNs or FAM-HLPNs), CD31 (green, blood vessels), and DAPI (blue, nuclei). For each group, six tissue sections were analyzed per mouse (*n* = 6 sections), with three biological replicates (*n* = 3 mice per group). *p < 0.05. **p < 0.01. ***p < 0.001 [one-way analysis of variance (ANOVA)].

Similarly, in the VEGFko model, which has infiltrative growth patterns (Figure 7), the selective accumulation of LinTT1-HLPNs was also obtained (Figure 7B,C). These results further endorse that LinTT1-HLPNs may cross the BBB, which still represents the main challenge in brain drug delivery and then accumulate in the tumor microenvironment.

In both tumor models, no fluorescence signal was detected in tissues treated with nontargeted FAM-HLPNs. The quantitative analyses showed that the fluorescent signal from FAM-HLPNs was comparable to that of the PBS control (Figures 6B and[Fig fig7]B). Conversely, LinTT1-HLPNs had significantly higher accumulation, with over double fluorescence intensity in the wtGBM model and approximately double fluorescence intensity in the VEGFko model (Figures 6B and[Fig fig7]B). These results highlighted the effective multistep GBM targeting properties of LinTT1-HLPNs, allowing selective delivery to GBM by overcoming biological barriers.^65,75^

In both the infiltrative and noninfiltrative glioblastoma models, untargeted nanoparticles also had hepatic accumulation, demonstrating their physiological metabolism and clearance. These results (Figures S6 and S7) endorse the expected biodistribution of untargeted HPLNs.

Targeted nanomedicine represents a promising strategy to enhance the precision and effectiveness of cancer treatment, particularly for challenging tumors such as GBM. In this context, we developed a peptide-functionalized nanocarrier system designed to selectively target GBM cells and their microenvironment. LinTT1-HLPNs exhibited favorable physicochemical characteristics and demonstrated long-term stability. Functionalization with the LinTT1 peptide markedly enhanced nanoparticle interaction with GBM cells, leading to a greater *in vitro* cytotoxic effect of encapsulated TMZ compared to the free drug. *In vivo* studies further endorsed the selective accumulation of LinTT1-HLPNs in both invasive and noninvasive GBM models. Collectively, these results support the potential of LinTT1-HPLNs as a targeted nanoplatform for precision GBM therapy.

## 2 Methods

### 2.1 Preparation of HLPNs

HLPNs were prepared by a single-step nanoprecipitation method as reported by Zhang et al., with some modification.^50^ Briefly, PLGA was dissolved in acetonitrile to a final concentration of 20 mg/mL (phase A). The lipid mixture, consisting of Lipoid S100, DSPE-PEG-2,000, and DSPE-PEG-2,000-maleimide (molar ratio 7:2:1), was dissolved in 400 μL of ethanol and then added to prewarmed water (65 °C) to have a final volume of 10 mL (phase B). 250 μL of phase A was added dropwise under continuous stirring (600 rpm) to 2.25 mL of phase B, thus having a final lipid-to-polymer ratio of ∼20%.

The resulting HPLNs were sonicated in a water bath for 10 min followed by stirring at room temperature for 2 h to have nanoparticle formation and organic solvent evaporation. To remove free molecules and organic solvent residuals, the HLPNs were centrifuged at 8,000 rpm for 5 min using Amicon Ultra-15 filters. The HLPNs were then collected, washed twice with 1 mL of phosphate-buffered saline (PBS, pH 7.4), and finally resuspended in PBS (pH 7.4) for further analyses.

When required, TMZ was dissolved in phase A before mixing with phase B. Three different concentrations of TMZ were used for HLPNs@TMZ preparation, i.e., 1, 2, and 4 mg/mL. HLPNs@TMZ, with the best results of drug loading and entrapment efficiency, were used for further studies.

After the purification, RPAR or LinTT1 peptides were conjugated to the surface of HPLNs as previously described,^88^ to have empty targeted nanoparticles, i.e., RPAR-HLPNs and LinTT1-HLPNs, or TMZ-loaded targeted nanoparticles, i.e., LinTT1-HLPNs@TMZ, respectively. scrRPAR- and FAM-conjugated HPLNs were used as a control for RPAR- and LinTT1-HLPNs, respectively.

### 2.2 Physicochemical Characterization of HLPNs

The average diameter, polydispersity index (PDI), and zeta potential were measured using a Zetasizer Ultra (Malvern Panalytical, UK). Samples were diluted 50-fold with either phosphate-buffered saline (PBS) or Milli-Q water to prevent multiscattering phenomena. Measurements were conducted at 25 °C, and results were expressed as the mean of three independent experiments ± standard deviation (S.D.). The dynamic light scattering (DLS) analysis was carried out according to the setup previously published.^89^

Transmission electron microscopy (TEM) analyses were performed to further study sizes and morphology of nanoparticles.^90^ Samples were negatively stained using uranyl acetate (2 wt %) and left to dry on a coated grid. Images were acquired through a Veleta digital camera (Olympus Soft Imaging System), by operating at 100 kV (Tecnai G2 (FEI) transmission electron microscope) as previously published.^91^

Successful conjugation of the peptide or FAM group on the surface of HLPNs was studied by measuring the fluorescence of the 5-carboxyfluorescein (5-FAM) group in the peptides’ backbone (RPAR, scrRPAR, and LinTT1) or directly conjugated to the HLPN surface (FAM-HLPNs). The quantification of the peptide on the HLPN surface was carried out after the purification process by using an external calibration curve (*y* = 0.003*x* + 0.0013, *R* ^2^ = 0.995) through a Nanodrop 2000c spectrophotometer (Thermo Scientific Inc., USA).

The stability of the LinTT1-HLPNs@TMZ was tested over a period of 4 weeks at +4 °C. At specific time points (7, 14, 21, and 28 days), samples were allowed to equilibrate to room temperature and then analyzed by using DLS.

To study the stability of LinTT1-HLPNs@TMZ in human plasma, 200 μL of HLNPs were incubated with 1 mL of human plasma (50% v/v) and maintained at 37 °C under gentle magnetic stirring for up to 72 h. PBS (10 mM) was used as a control. At fixed time points (15, 30, and 45 min, 1, 2, 4, 6, 8, 24, 48, and 72 h), 50 μL of samples were withdrawn and analyzed by using the Zetasizer Ultra (Malvern Panalytical, UK).

### 2.3 TMZ Entrapment Efficiency, Drug Loading, and Release Kinetic Profile

The drug loading percentage (D.L.%) and the entrapment efficiency percentage (E.E.%) were calculated using an indirect method. Briefly, after purification of HLPNs, the amount of drug in the supernatant was removed by using Amicon filters (cutoff 100 kDa) and then quantified by HPLC analysis.

The analysis was performed using an HPLC system (ThermoFisher Scientific) equipped with a quaternary pump, autosampler, thermostated column compartment, and UV–vis detector. A C18 GreatSmart column (4.6 × 150 mm, 5 μm) was used for the separation, and the column temperature was maintained at 40 °C. The mobile phase consisted of an aqueous solution of acetic acid (0.5% v/v) as phase A and acetonitrile as phase B. TMZ analysis was carried out using a linear gradient: from 5% to 45% of phase B over 10 min followed by a return from 45% to 5% of phase B in 1 min. The flow rate was set at 1 mL/min, and the total run time was 14 min. TMZ was quantified at maximum absorbance wavelength of 329 nm by using a proper external calibration curve (*y* = 0.0627*x* + 0.0106, *R* ^2^ = 0.9991).

EE% and DL% were then calculated as following reported:

$$\mathrm{EE}\%=\frac{\left(D_{\mathrm{tot}}-D_{\mathrm{sur}}\right)}{D_{\mathrm{tot}}}\times 100$$ 1

$$\mathrm{DL}\%=\frac{\left(D_{tot}-D_{sur}\right)}{\left(L+Pw\right)}\times 100$$ 2

where *D*_tot_ is the total amount of drug added during preparation, *D*_sur_ is the drug in the supernatant, and *L* + *Pw* is the amount of lipids and PLGA used to make HLPNs.

The release kinetic studies were carried out by using bag dialysis method as previously described with minor modification.^70^ Briefly, 1 mL of LinTT1-HLPNs@TMZ were placed into a dialysis bag (Spectra/Por 1 Standard RC Dry Dialysis Tubing, 12–14 kDa, Spectrum Laboratories, USA) and held in a backer with PBS at pH 7.4 or 5.5. To maintain the sink conditions, the receptor medium was maintained under continuous stirring at 200 rpm, and 1 mL of receptor medium was withdrawn at specific time points (30 min, 1, 2, 4, 6, 8, 24, 48, and 72 h) and replaced with the same volume of fresh medium. The amount of drug was quantified by using HPLC method previously reported and the release percentage (%) was calculated as following reported:

$$\mathrm{release}\%=\left[\frac{D_{\mathrm{rel}}}{D_{\mathrm{en}}}\times \mathrm{dl}\right]\times 100$$ 3

where *D*_rel_ is the amount of released drug, *D*_en_ is the amount of drug entrapped into HLPNs, and dl is the dilution factor.

For the release study, both TMZ and AIC were quantified. TMZ was quantified as reported above, while AIC was quantified using the same linear gradient at wavelength of 263 nm by using a proper external calibration curve (*y* = 0.858*x* – 0.1809, *R* ^2^ = 0.9968).

### 2.4 Cell-free Binding Study

For cell-free binding assay, Hunt’s protocol was followed with some modifications.^32^ Ni-NTA magnetic agarose beads (Qiagen, Germany) were coated with the p32 protein or b1b2 domain of NRP-1­(15 μg of protein/10 μL of beads). The binding buffer consisted of 0.05% Tween-20 and 5 mM imidazole in PBS pH 7.4 and 50 mM Tris-buffer pH 7.5 supplemented with 1 M NaCl, 5 mM imidazole, and 0.05% NP40 (Igepal CA-630, Thermo Scientific Inc., USA) for p32 and NRP-1, respectively. Fluorescently labeled HPLNs were incubated with the p32- or NRP-1-coated beads at room temperature for 1 h, with or without the addition of serine protease uPA. After incubation, the beads were washed with their respective binding buffers supplemented with 1% (v/v) BSA and then eluted. The elution was performed by using 400 mM imidazole in PBS pH 7.4 or 400 mM imidazole, 300 mM NaCl, 0.05% NP40, and 1% BSA in PBS (pH 7.4) for p32-coated beads and NRP-1-coated beads, respectively. The fluorescence of the eluted samples was quantified using a fluorescence plate reader (FlexStation II, Molecular Devices, CA, USA).

### 2.5 Uptake Studies

The uptake of RPAR-HLPNs or scrRPAR-HLPNs in PPC-1 and M21 cells was studied as previously published by d’Avanzo et al.^70^ Briefly, cells were seeded in 12-well plates with coverslips (12 mm diameter, Marienfeld-Superior) at a density of 100,000 cells/well and incubated for 24 h (37 °C and 5% CO_2_). The following day, cells were incubated with RPAR-HLPNs or scrRPAR-HLPNs, at a lipid concentration of 0.25 mg/mL. After 1 h of incubation, the cells were washed with fresh PBS, fixed with 4% (w/v) paraformaldehyde (PFA) for 10 min at room temperature, and counterstained with DAPI (500 μL, 1 μg/mL, Thermo Scientific Inc., USA). The coverslips were mounted with 20 μL of mounting medium (Fluoromount-G; Electron Microscopy Sciences) and sealed with a nail polish. Confocal images were acquired using an Olympus FV1200MPE confocal microscope (Olympus, Japan) with an UPlanSApo 60×/1.35na objective (Olympus, Japan) and analyzed using FluoView FV10-ASW Ver.4.2a Viewer software.

For flow cytometry analysis, PPC-1 and M21 cells were seeded in a 24-well plate and the next day were treated with RPAR-HLPNs or scrRPAR-HLPNs at a lipid concentration of 0.125 mg/mL. After 1 h of incubation, cells were washed with PBS, detached with enzyme-free dissociation buffer, harvested, and transferred to 1.5 mL tubes. The collected cells were centrifuged (250*g* at room temperature for 5 min), resuspended in 300 μL of PBS, and analyzed by using a BD Accuri C6 Plus flow cytometer (BD Biosciences, New Jersey, USA).

The selectivity of RPAR binding to the b1b2 domain of Neuropilin 1 was further studied through binding assay in which cells were preincubated with blocking antibody (mAb7E8) and no-binding pocket antibody mAb3E7. Briefly, PPC-1 cells were seeded as described earlier and treated with mAb7E8 or mAb3E7 antibodies at final concentrations of 0.010 and 0.020 mg/mL followed by 2 h of incubation at 37 °C. The cells were then treated with RPAR-NSVs or scrRPAR-NSVs for 1 h. After incubation, cells were washed, detached, and analyzed by flow cytometry analysis and confocal microscopy, as described above.

### 2.6 Induction of p32 Exposure on the Cell Membrane of GMB Cells and Uptake Studies

Wt-GBM and VEGF-ko-GBM cell lines were cultured in MEM with Earle’s salts (Capricorn Scientific, Germany) supplemented with 100 IU/mL of penicillin/streptomycin, 1% (v/v) sodium pyruvate, 0.01 M HEPES, 0.6% (v/v) glucose (Applichem, USA), and 5% (v/v) heat-inactivated FBS (GE Healthcare, UK).

The GL261 cell line was cultured in DMEM supplemented with 100 IU/mL of streptomycin and penicillin and 10% (v/v) fetal bovine and 1% glutamine. The expression of p32 protein on all GBM cell membranes was assessed under normal and stress conditions. To mimic *in vivo* stress conditions, an in-house optimized protocol was used where cells (80% of confluence) were incubated with a glucose- and pyruvate-reduced culture medium for 24 h. These conditions ensured a cell survival rate of 98% (data not shown).

For the staining of p32 protein, cells were detached and then centrifuged at 150*g* for 5 min at 4 °C. The supernatant was withdrawn, and the cells were resuspended in PBS containing 1% (v/v) BSA to achieve a final concentration of 700,000 cells in 500 μL. The cells in suspension were then incubated with a primary antibody, rabbit anti-p32 (in-house developed; dilution 1:100, stock concentration 4.5 mg/mL), for 1 h at 4 °C under slow rotation. Following this incubation, the cells were centrifuged at 250*g* for 5 min at +4 °C, the supernatant was removed, and the cells were resuspended in 500 μL of PBS with 1% (v/v) BSA. This washing and centrifugation step was repeated three times. Subsequently, the cells were incubated with a secondary antibody, goat antirabbit Alexa Fluor 488 (dilution 1:1000 v/v), in PBS supplemented with 1% (v/v) BSA for 45 min at +4 °C. The cells were purified through three cycles of centrifugation/wash and were resuspended in 500 μL of PBS with 1% (v/v) BSA. The cells were suspended in 300 μL of PBS and analyzed using a BD Accuri C6 Flow Cytometer for fluorescence detection.

For the uptake studies, 600,000 wt-GBM, VEGF-KO-GBM, or GL261 cells in suspension were slowly rotated at 37 °C in the presence of 0.125 mg/mL of LinTT1-HLPNPs or FAM-HLPNs in PBS supplemented with 1% (v/v) BSA for 30 min. After incubation, the cells were washed twice with PBS/BSA. The collected cells were resuspended in 300 μL of PBS and analyzed by using a BD Accuri C6 Plus flow cytometer (BD Biosciences, New Jersey, USA).

### 2.7 Cytotoxicity Studies

Wt-GBM and VEGFko-GBM cells were subjected to 24 h of nutrient-reduction stress prior to the assay, whereas GL261 cells, which intrinsically express high levels of the p32 receptor, were maintained under normal conditions. All cell lines were seeded into 96-well plates at a density of 10,000 cells/well and allowed to attach overnight. The cells were then treated with free TMZ, empty-HLPNs, LinTT1-HLPNs@TMZ, and FAM-HLPNs@TMZ at various drug concentrations (2.5, 5, 10, 25, and 50 μM). After 30 min of incubation, cells were washed with fresh PBS to eliminate any free TMZ or HLPNs@TMZ that has not been internalized by the cells. Fresh complete medium was then added, and cells were incubated for an additional 72 h.

Afterward, 10 μL of MTT reagent (5 mg/mL) was added to each well. After 2 h of incubation, the resulting formazan product was quantified by measuring the absorbance at 540 nm using a Tecan Sunrise microplate reader (Tecan, Switzerland). Cell viability was calculated using the following equation:

$$\mathrm{cell}\;\mathrm{viability}(\%)=\frac{{\mathrm{Abs}}_{T}}{{\mathrm{Abs}}_{C}}\times 100$$ 4

where Abs_T_ was the absorbance of treated cells while Abs_C_ was the absorbance of untreated cells (control).

### 2.8 *In Vivo* Studies

To assess *in vivo* accumulation and binding efficiency of RPAR-HLPNs, preliminary studies were carried out in healthy nude mice.^92^ Mice were randomly assigned to three groups and intravenously injected via the tail vein with RPAR-HLPNs, scrRPAR-HLPNs, or PBS (negative control) at a dose of 0.072 mg of nanoparticles/g of mice body weight.

1 h after the injection, the mice were perfused with 20 mL of PBS via the left ventricle to remove unbound nanoparticles. The lungs were collected as target and control tissue, embedded in an optimal cutting temperature (OCT) compound (Leica), frozen in liquid nitrogen, and stored at −80 °C for further analysis.

### 2.9 *In Vivo* Binding Studies in GMB Mice Models

Prior to tumor cell implantation, mice were anesthetized via intraperitoneal injection. During the procedure, an ophthalmic gel was applied to protect the eyes from dehydration.

To induce GBM in the brain, a midline incision was made to expose the scalp. Tumor cells were stereotactically injected into the right striatum using a Hamilton syringe (7 × 10^5^ cells in 2.5 μL of PBS). After the injection, the cranial hole was sealed with bone wax, and the scalp incision was sutured. Mice were monitored daily and used for HPLN administration 6–7 days postimplantation of wtGBM cells or 10–12 days postimplantation of VEGFko cells.^75^

To assess HPLN accumulation and binding *in vivo*, we randomly divided mice into three groups. Each group received either LinTT1-HLPNs, FAM-HLPNs, or PBS as a negative control. HPLNs were administered intravenously via the tail vein at a dose of 0.072 mg of nanoparticles/g of mice body weight.

LinTT1-HLPNs, FAM-HLPNs, or PBS were allowed to circulate for 3 h, after which mice were perfused through the left ventricle with 20 mL of PBS. The brain was collected, embedded in an optimal cutting temperature (OCT) compound (Leica), and rapidly frozen in liquid nitrogen and stored at −80 °C until further analysis.

### 2.10 Cryosection Preparation and Immunostaining Analyses

Tissue cryosections (20 μm thick) were prepared using a cryostat and mounted onto Superfrost+ slides (Thermo Scientific, Waltham, MA, USA). The slides were stored at −20 °C until use.

For immune-staining, sections were equilibrated to room temperature, fixed with 4% v/v paraformaldehyde (PFA) in PBS for 15 min, and washed with PBST (PBS containing 0.05% v/v Tween-20) (Sigma-Aldrich, St. Louis, MO, USA). The cell membrane was permeabilized by using PBS with 0.2% v/v Triton X-100 (AppliChem, Darmstadt, Germany) at room temperature for 15 min followed by extra washes with PBST.

Blocking was conducted using a buffer containing 5% w/v BSA, 5% v/v goat serum, and 0.3 M glycine in PBST at room temperature for 1 h. Primary antibodies, diluted in an antibody solution (0.5% w/v BSA, 0.5% v/v goat serum in PBST), were applied and incubated overnight at 4 °C. Secondary antibodies were diluted in the same buffer and incubated with the sections at room temperature for 1 h. Following incubation, sections were washed and counterstained with DAPI (1 μg/mL in PBS) for 5 min at room temperature. LinTT1-HLPNs or FAM-HLPNs were visualized using a rabbit antifluorescein/Oregon Green antibody (1:200 v/v dilution) (Thermo Fisher Scientific, Invitrogen, USA) followed by a goat antirabbit Alexa Fluor 546 secondary antibody (1:400 v/v dilution), which amplifies the signal of the nanoparticles visualized in red (Thermo Fisher Scientific, Invitrogen, USA). The endothelium of blood vessels was labeled with a rat antimouse CD31 antibody (1:200 v/v dilution) (BD Biosciences, USA) and a goat antirat Alexa Fluor 488 secondary antibody (1:400 v/v dilution), which amplifies the signal visualized in green (Thermo Fisher Scientific, Invitrogen, USA).

Coverslips were mounted using Fluoromount-G, and imaging was performed using an Olympus FV1200MPE confocal microscope (Olympus Europa SE & Co. KG, Hamburg, Germany).

### 2.11 Statistical Analysis and *In Vivo* Image Quantification

Data is presented as mean ± standard deviation (SD) from three independent experiments. Statistical significance was determined using one-way ANOVA. *p*-values of <0.05, < 0.01, and <0.001 were considered statistically significant.

Fluorescence signals from *in vivo* experiments were quantified using ImageJ (NIH, Bethesda, MD). Regions of interest were manually selected, and the mean fluorescence intensity was calculated after background subtraction.

### 2.12 Animals

Animal experiments were conducted in accordance with protocols approved by the Animal Experimentation Committee of the Ministry of Agriculture. Athymic nude mice (Hsd/Athymic Fox1 nu Harlan) were purchased from Envigo (Netherlands) and maintained under standard housing conditions of the Animal Facility of the Institute of Biomedicine and Translational Medicine, University of Tartu (Tartu, Estonia) (Animal permit #299).

## Supplementary Material



## Notes

Materials; Table S1: Physicochemical characterization of HLPNs; Table S2: Physicochemical characterization of LinTT1-HLPNs@TMZ incubated in human plasma (50% v/v) at 37 °C; Figure S1: Stability profiles of FAM-HLPNs@TMZ incubated with PBS buffer; Figure S2: Fitting drug release profile with different kinetic models; Table S3: Fitting (R^2^) of mathematical models with release profile of TMZ and AIC; Table S4: Physicochemical characterization of RPAR-HLPNs and scrRPAR-HLPNs; Figure S3: Cellular uptake studies; Table S5: Statistical analysis of cytotoxic studies; Figure S4: *In vivo* homing studies of peptide targeted HLNPs to lung tissue; Figure S5: *In vivo* biodistribution of untargeted HLPNs; Figure S6: *In vivo* hepatic accumulation of untargeted HLPNs in VEGFko-GBM tumor-bearing mice; Figure S7: In vivo hepatic accumulation of untargeted HLPNs in wtGBM tumor-bearing mice (PDF)

## ABBREVIATIONS

AIC: 5-aminoimidazole-4-carboxamide
Bare-HLPNs: nonconjugated HLPNs
CendR: C-end rule peptide
FAM: 6-carboxyfluorescein
FAM-HLPNs: FAM-labeled HLPNs
HLPNs: hybrid lipid–polymer nanoparticles
LinTT1: linear TT1 peptide
LinTT1-HLPNs: LinTT1-functionalized HLPNs
LinTT1-HLPNs@TMZ: LinTT1-HLPNs loaded with temozolomide
MMR: mismatch repair
NRP1: neuropilin-1
PDI: polydispersity index
RPAR-HLPNs: RPAR-functionalized HLPNs
scrRPAR-HLPNs: scrambled RPAR-functionalized HLPNs
TMZ: temozolomide
