# Supplementary material for: LinTT1-Functionalized Hybrid Lipid–Polymer Nanoparticles for Glioblastoma Targeting
Source: ACS Pharmacol Transl Sci. 2025 Sep 30;8(10):3654–68. doi: 10.1021/acsptsci.5c00537 (PMC12519261; doi:10.1021/acsptsci.5c00537)
Supplement: Supplementary file 1 [file pt5c00537_si_001.pdf]

SUPPORTING INFORMATION

LinTT1 – functionalized hybrid lipid – polymer nanoparticles for glioblastoma targeting

Antonella Rocchi[a,b,1], Valeria Sidorenko[a,n,1], Nicola d'Avanzo[c,d,1], Luca Marchetti[a], Jhalak Sethi[a], Luigi Ciriolo[e], Anna Maria Tolomeo[f,g], Maria Grazia Cifone[h], Paola Palumbo[h], Massimo Fresta[d,e], Tambet Teesalu[a,i,*], Christian Celia[b,l,m,*]

[a]*Laboratory of Precision and Nanomedicine, Institute of Biomedicine and Translational Medicine, University of Tartu, Ravila 14b, 50411 Tartu, Estonia.*

[b]*Department of Pharmacy, University of Chieti – Pescara "G. d'Annunzio", Via dei Vestini 31, 66100 Chieti, Italy.*

[c]*Department of Clinical and Experimental Medicine, University of Catanzaro "Magna Græcia", V.le "S. Venuta", Catanzaro, I-88100, Italy.*

[d]*Research Center "ProHealth Translational Hub", Department of Experimental and Clinical Medicine, "Magna Graecia" University of Catanzaro, Campus Universitario "S. Venuta" – Building of BioSciences, Viale S. Venuta, I-88100 Catanzaro, Italy.*

[e]*Department of Health Science, University of Catanzaro "Magna Græcia", V.le "S. Venuta", Catanzaro, I-88100, Italy.*

[f]*Department of Cardiac, Thoracic and Vascular Science and Public Health, University of Padova, I-35128, Padua, Italy.*

[g]*Perdiatric Research Institute "Città della Speranza", Corso Stati Uniti, 4, I-35127, Padua, Italy*

[h]*Department of Life, Health & Environmental Sciences, University of L'Aquila, Via Pompeo Spennati, Building Rita Levi Montalcini, Coppito, I-67100 L'Aquila, Italy.*

[i]*Materials Research Laboratory, University of California, Santa Barbara, 93106 California, United States.*

[l]*Institute of Nanochemistry and Nanobiology, School of Environmental and Chemical Engineering, Shanghai University, 200444 Shanghai, China.*

[m]*SCM Nutraceutici Universitari Srl, Strada degli Oliveti 73, I-66100 Chieti, Italy.*

[n]*Division of GI/Endocrine Surgery, Department of Surgery, Columbia University Irving Medical Center, New York, NY 10032, USA.*

[1]*These authors equally contributed*

**Corresponding authors:*

Tambet Teesalu, email: tambet.teesalu@ut.ee; Christian Celia, email: c.celia@unich.it

Table of Contents

Materials	S-3
Table S1. Physicochemical characterization of HLPNs	S-4
Table S2. Physicochemical characterization of LinTT1-HLPNs@TMZ incubated in human plasma (50% v/v) at 37°C	S-5
Figure S1. Stability profiles of FAM-HLPNs@TMZ incubated with PBS buffer	S-6
Figure S2. Fitting drug release profile with different kinetic models	S-7
Table S3. Fitting (R^2) of mathematical models with release profile of TMZ and AIC	S-8
Table S4. Physicochemical characterization of RPAR-HLPNs and scrRPAR-HLPNs.	S-9
Figure S3. Cellular uptake studies	S-10
Table S5. Statistical analysis of cytotoxic studies	S-12
Figure S4. *In vivo* homing studies of peptide targeted HLNPs to lung tissue	S-15
Figure S5. *In vivo* biodistribution of untargeted HLPNs	S-16
Figure S6 In vivo hepatic accumulation of untargeted HLPNs in VEGFko-GBM tumor-bearing mice	S-17
Figure S7 In vivo hepatic accumulation of untargeted HLPNs in wtGBM tumor-bearing mice.	S-18

MATERIALS

N-(carbonyl-methoxypolyethylene glycol-2000)-1,2-distearoyl-sn-glycero-3-phosphoethanolamine (DSPE-mPEG2000) and 1,2-distearoyl-sn-glycero-3-phosphoethanolamine-N-[maleimide(polyethylene glycol)-2000] (DSPE-PEG2000-Mal) were purchased from Avanti Polar Lipids (Merck, Italy). Poly(D,L-lactide-co-glycolide) (PLGA, lactide: glycolide ratio 50: 50), Temozolomide ≥98% (HPLC), powder and 4-Amino-5-imidazolecarboxamide hydrochloride, AICA 98%, Paraformaldehyde (PFA) and human plasma, Tween 20, (4,5-dimethylthiazol-2-yl)-2,5-diphenyltetrazolium bromide (MTT) reagent were obtained from Sigma-Aldrich (Merck, Italy). Lipoid S100 was a kindly gift of Lipoid (Germany). Amicon Ultra 0.5 mL centrifugal filters with a molecular weight cut-off (MWCO) of 100 kDa were obtained from Millipore (Sigma, USA). Agarose beads were obtained from Qiagen GmbH (Hilden, Germany). Triton X-100 was obtained from AppliChem (Darmstadt, Germany). Dulbecco's Modified Eagle Medium (DMEM) and phosphate-buffered saline (PBS, pH 7.4) were obtained from Lonza (Belgium). Minimum Essential Medium (MEM) with Earle's salts was purchased from Capricorn Scientific (Germany) and supplemented with 100 IU/mL penicillin/streptomycin, 1% sodium pyruvate, 0.01 M HEPES, 0.6% glucose (Applichem, USA), and 5% heat-inactivated fetal bovine serum (FBS) (GE Healthcare, UK). Trypan Blue was obtained from Gibco (Thermo Fisher Scientific, USA). Bovine serum albumin (BSA) and fetal bovine serum (FBS) were purchased from Capricorn Scientific (Germany). For immune-staining, goat serum (GE Healthcare, UK) was used as a blocking agent for 1 hour. Primary antibodies included rabbit anti-fluorescein (Thermo Fisher Scientific, MA, USA) and rat anti-mouse CD31 (BD Pharmingen, USA). Secondary antibodies were Alexa Fluor 488-conjugated goat anti-rabbit IgG and Alexa Fluor 546-conjugated goat anti-rabbit IgG (both from Invitrogen, Thermo Fisher Scientific, USA). The peptides, i.e. FAM-Cys-(Ahx)-RPARPAR-OH (RPAR); FAM-Cys-(Ahx)-RRAAPRP-OH (scrambled peptide or scrRPAR); FAM-Cys-(Ahx)-AKRGARSTA-NH2 (LinTT1); FAM-Cys-(Ahx)-NH2 (FAM), where Cys is cysteine, FAM is 5-carboxyfluorescein, and Ahx is an aminohexanoic acid linker were purchased from TAG Copenhagen (Denmark).

PPC-1 (Human primary prostate adenocarcinoma) and M21 (Human melanoma) were kindly provided by Erkki Ruoslahti laboratory at Research Center Sanford Burnham Prebys Medical Discovery Institute and by prof. David Cheresh at University of California San Diego (USA), respectively. GL261 (Mouse Glioma 261) was donated by I.R.C.C.S. Ospedale San Raffaele (Italy). wtGBM (wild type Mouse glioblastoma) and VEGFko-GBM (VEGF knockout Mouse glioblastoma) were obtained from Gabriele Bergers (Leuven, Belgium)

All other reagents and chemicals used in this study were of analytical grade and were used without further purification.

SUPPLEMENTARY RESULTS

Table S1. Physicochemical characterization of HLPNs.

Samples	Mean Size (nm)	Zeta Potential (mV)	PDI	Particles concentration (n° particles/mL)
**HLPNs@TMZ**	152±1	-32.5±1.4	0.053±0.04	5E+11 ± 4.6E+10
**LinTT1-HLPNs@TMZ**	173±2	-27.6±0.9	0.03±0.02	3.3E+11±1.1E+10
**FAM-HLPNs**	157±1	-33,7±1.9	0.06±0.03	5.8E+11±3E+10
**FAM-HLPNs@TMZ**	164±1	-32,6±1.2	0.04±0.02	4E+11±9.5E+09

Table S2. Physicochemical characterization of LinTT1-HLPNs@TMZ incubated in human plasma (50% v/v) at 37°C.

Incubation time	Mean Size (nm)	PDI	Zeta Potential (mV)
After 15 min	163.8±21.9	0.1	-25.6±0.2
After 30 min	171.8±7.7	0.1	-26.2±1.2
After 1 h	169.6±17.7	0.1	-27.3±3.6
After 2 h	161.8±27.4	0.1	-25.1±2.5
After 4 h	176.5±7.4	0.14	-25.6±2.7
After 6 h	188.5±5.6	0.1	-22.6±4.6
After 8 h	192.5±6.3	0.3	-23.7±3.9
After 24 h	269±150.2	0.6	-11.9±1.3
After 48 h	502.3±219.8	0.55	-11.6±3.2
After 72 h	698.8±212.9	0.6	-9.4±0.9

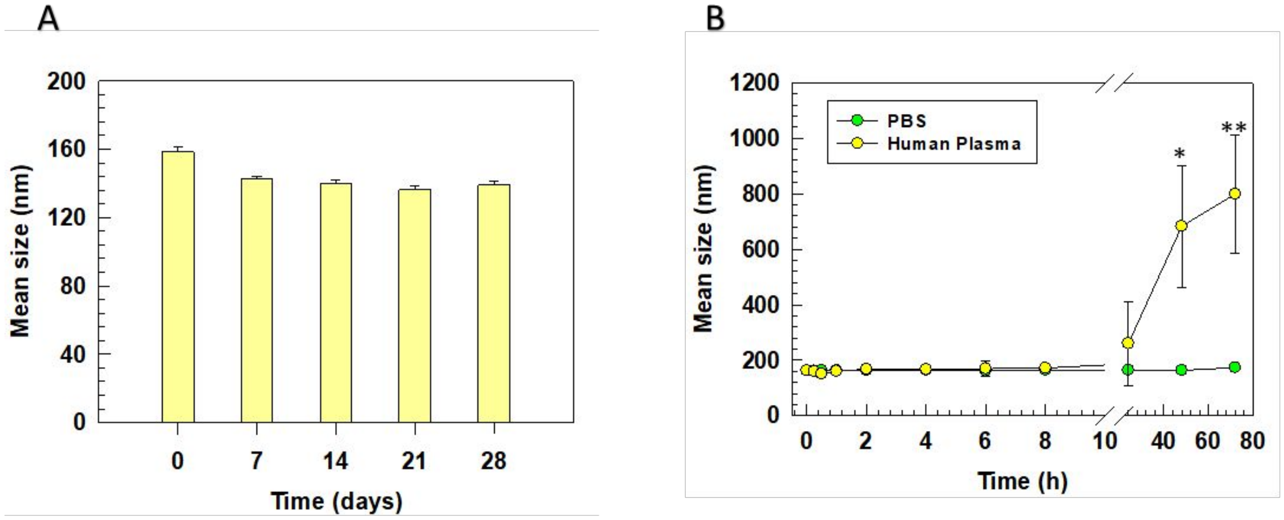

Figure S1. Stability profiles of FAM-HLPNs@TMZ incubated with PBS buffer (A) and human plasma at 37 °C **(B)**. Results are the mean of three independent experiments ± standard deviation (S.D.) *p < 0.05. **p < 0.01. ***p < 0.001 [one-way analysis of variance (ANOVA)].

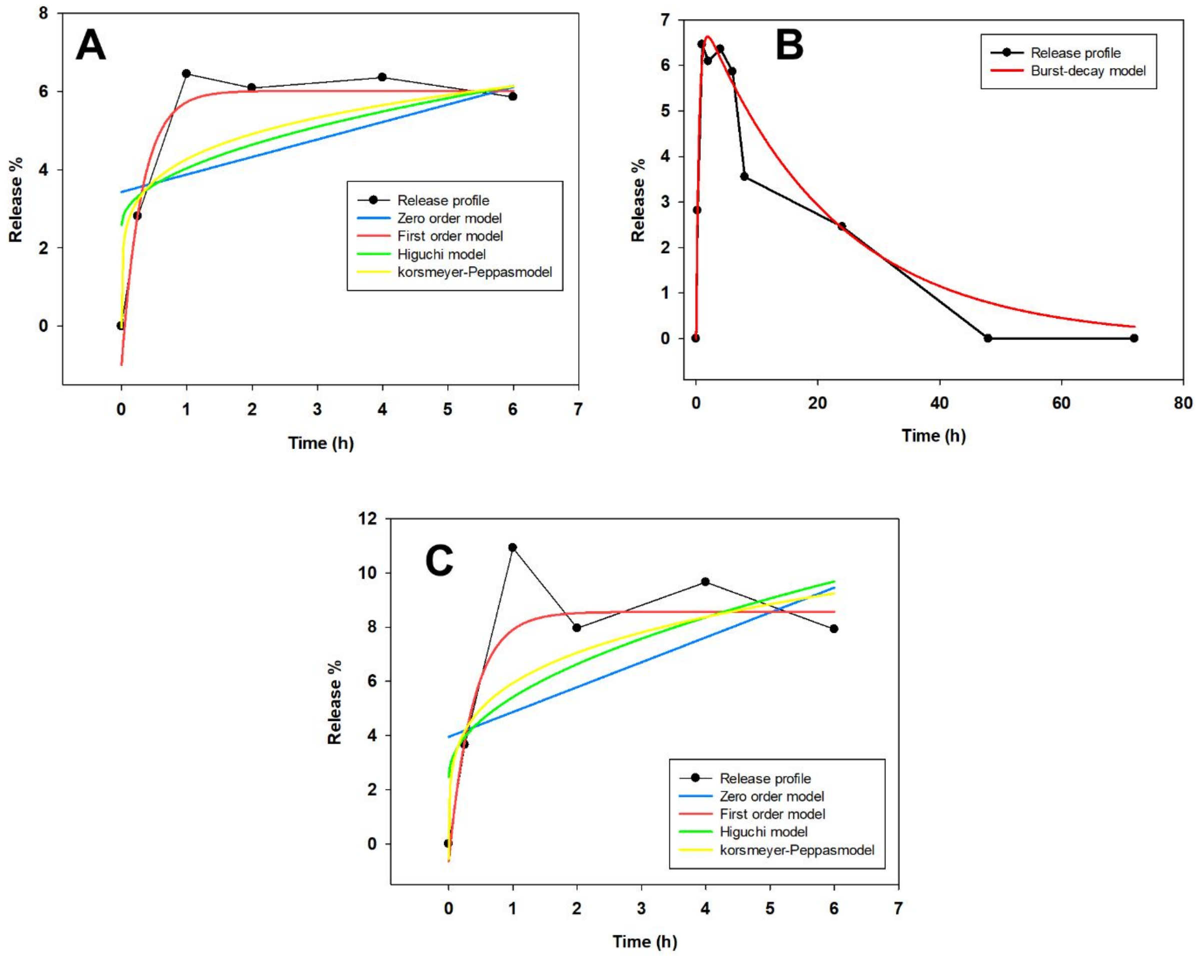

Figure S2. Fitting drug release profile with different kinetic models. **(A)** shows the fitting of TMZ release at pH 5.5 with First order, zero order, Higuchi and Korsmeyer-Peppas models within the incubation time 0-6 h, while **(B)** shows the fitting of TMZ profile at pH 5.5 up to 72 h with the Burst-decay model. **(C)** shows the fitting of AIC release at pH 7.4 with First order, zero order, Higuchi and Korsmeyer-Peppas models within the incubation time 0-6 h. Constant offset term C was applied for the used kinetic models.

Incorporating offset terms improved models' alignment with experimental data and reflected realistic release behavior, as also emphasized by Mircioiu et al. (2019) in their discussion on modeling best practices (10.3390/pharmaceutics11030140).

Table S3. Fitting (R^2) of mathematical models with release profile of TMZ and AIC

Mathematical Models	TMZ Release pH 5.5 (0-6 h)	AIC Release pH 7.4 (0-6 h)	TMZ Release pH 5.5 (0-72 h)
Zero-Order	0.33	0.19	---
First-Order	0.95	0.84	---
Higuchi	0.55	0.44	---
Korsmeyer-Peppas	0.80	0.61	---
Burst-Decay	---	---	0.94

Table S4. Physicochemical characterization of RPAR-HLPNs and scrRPAR-HLPNs.

Samples	Mean Size (nm)	Zeta Potential (mV)	PDI
**RPAR-HLPNs**	160±3	-30.4±2.1	0.061±0.05
**scrRPAR-HLPNs**	158±4	-30.6±2.7	0.058±0.07

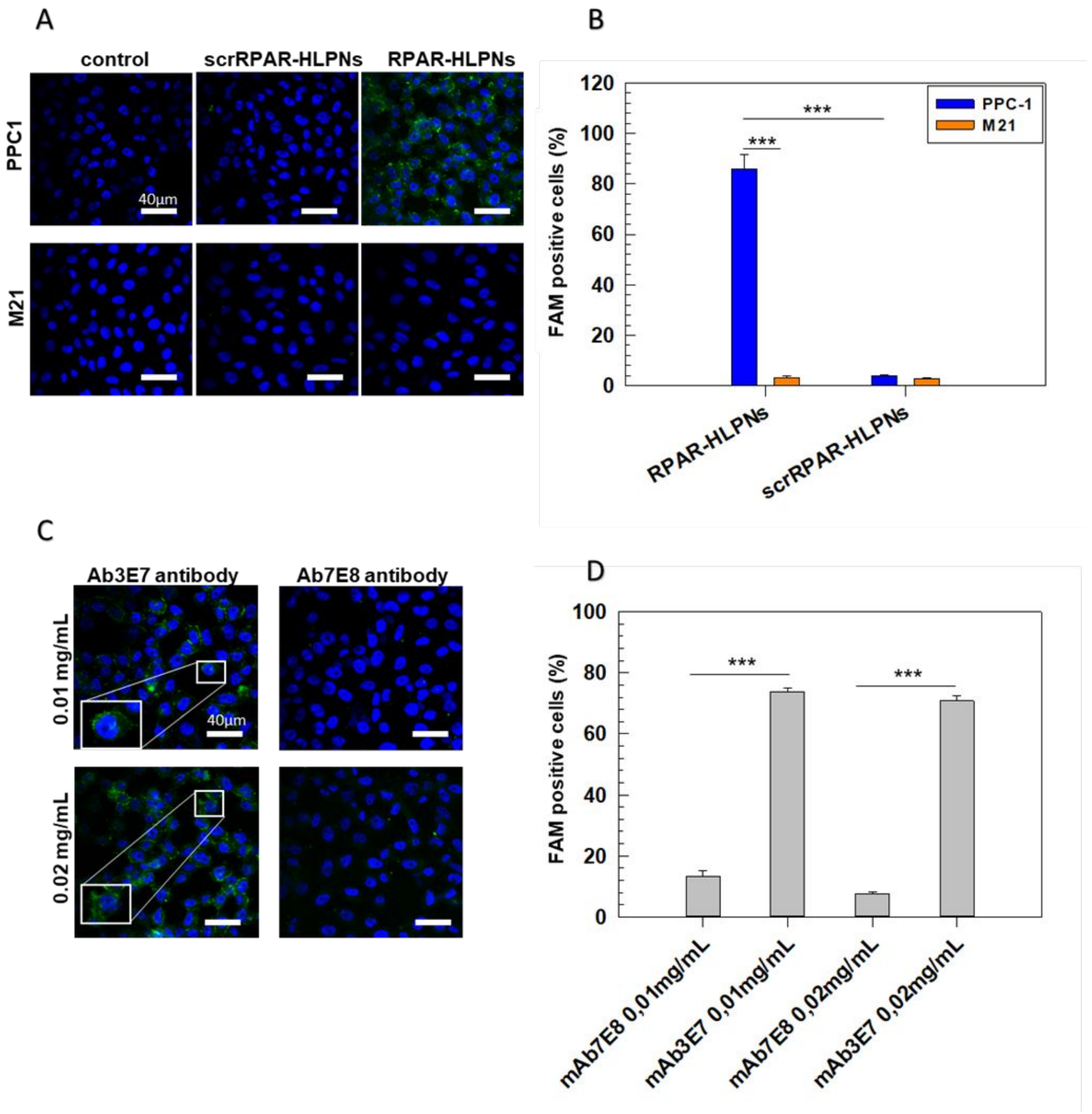

Figure S3. Cellular uptake studies. (A) Confocal laser scanning microscopy images showing NRP-1 dependent cellular uptake of RPAR-HLPNs. PPC-1 (NRP-1-positive) and M21 (NRP-1-negative) cells were incubated for 1 hour with either RPAR-HLPNs or scrRPAR-HLPNs at lipid concentrations of 0.250 mg/mL. FAM-labeled HLPNs were visualized via the Alexa Fluor 488 channel (green), and nuclei were counterstained with DAPI (blue). Significant intracellular fluorescence is observed in PPC-1 cells treated with RPAR-HLPNs, whereas M21 cells exhibit minimal uptake. Scale bar: 40 µm. **(B)** Quantitative analysis of cellular internalization of RPAR-HLPNs by flow cytometry. PPC-1 and M21 cells were incubated for 1 hour with RPAR-HLPNs or scrRPAR-HLPNs at lipid concentration of 0.125 mg/mL. Cellular uptake is expressed as the percentage of fluorescence-positive cells. Data represent mean ± standard deviation (S.D.) from three independent experiments

(n = 3), with 10,000 events recorded per condition. **(C-D)** NRP-1 CendR-binding pocket-dependent uptake of RPAR-HLPNs by PPC-1 cells. PPC-1 cells were pretreated for 2h with either a blocking antibody targeting the NRP-1 CendR-binding pocket (mAb7E8) or a non-blocking control antibody (mAb7E7) at concentrations of 0.01 and 0.02 mg/mL. Internalization of RPAR-HLPNs was then assessed following 1 hour of incubation. **(C)** cellular uptake was visualized by confocal laser scanning microscopy, using Alexa Fluor 488 (green) for FAM-labeled HLPNs and DAPI (blue) for nuclear staining. **(D)** Quantitative analysis of uptake was performed via flow cytometry, recording at least 10,000 events per condition. Data are presented as the percentage of fluorescence-positive cells and represent the mean ± standard deviation (S.D.) from three independent experiments. A significant reduction in RPAR-HLPNs internalization was observed in the presence of blocking mAb7E8 compared to mAb7E7. Results are the mean of three independent experiments ± standard deviation (S.D.) *$p < 0.05$. **$p < 0.01$. ***$p < 0.001$ [one-way analysis of variance (ANOVA)].

Table S5. Statistical analysis of cytotoxic studies. VEGFko-GBM, wtGBM and GL261 cells were treated with LinTT1-HLPNs@TMZ, FAM-HLPNs@TMZ and free TMZ at different TMZ concentrations in the range from 2.5 and 50 µM. *$p < 0.05$. **$p < 0.01$. ***$p < 0.001$ [one-way analysis of variance (ANOVA)].

		**wtGBM**	**VEGFko-GBM**	**GL261**
**TMZ CONCENTRATION (µM)**	**COMPARATIVE ANALYSIS**	**72H**	**72H**	**72H**
**2.5 µM**	HLPNs vs free TMZ	N.S.	N.S.	N.S.
	HLPNs vs LinTT1-HLPNs@TMZ	*	N.S.	N.S.
	HLPNs vs FAM-HLPNs@TMZ	N.S.	N.S.	N.S.
	free TMZ vs LinTT1-HLPNs@TMZ	*	N.S	N.S.
	free TMZ vs FAM-HLPNs@TMZ	N.S.	N.S.	N.S.
	LinTT1-HLPNs@TMZ vs FAM-HLPNs@TMZ	**	*	N.S.
**5 µM**	HLPNs vs free TMZ	N.S.	N.S.	N.S.
	HLPNs vs LinTT1-HLPNs@TMZ	***	N.S.	N.S.
	HLPNs vs FAM-HLPNs@TMZ	N.S.	N.S.	N.S.
	free TMZ vs LinTT1-HLPNs@TMZ	*	N.S.	N.S.
	free TMZ vs FAM-HLPNs@TMZ	N.S.	N.S.	N.S.

	LinTT1-HLPNs@TMZ vs FAM-HLPNs@TMZ	**	N.S.	N.S.
**10 µM**	HLPNs vs free TMZ	N.S.	N.S.	N.S.
	HLPNs vs LinTT1-HLPNs@TMZ	*	N.S.	N.S.
	HLPNs vs FAM-HLPNs@TMZ	N.S.	N.S.	N.S.
	free TMZ vs LinTT1-HLPNs@TMZ	**	N.S.	N.S.
	free TMZ vs FAM-HLPNs@TMZ	N.S.	N.S.	N.S.
	LinTT1-HLPNs@TMZ vs FAM-HLPNs@TMZ	**	N.S.	N.S.
**25 µM**	HLPNs vs free TMZ	N.S.	N.S.	N.S.
	HLPNs vs LinTT1-HLPNs@TMZ	***	N.S.	N.S.
	HLPNs vs FAM-HLPNs@TMZ	*	N.S.	N.S.
	free TMZ vs LinTT1-HLPNs@TMZ	***	N.S.	N.S.
	free TMZ vs FAM-HLPNs@TMZ	N.S.	N.S.	N.S.
	LinTT1-HLPNs@TMZ vs FAM-HLPNs@TMZ	**	***	*
**50 µM**	HLPNs vs free TMZ	N.S.	N.S.	N.S.

	HLPNs vs LinTT1-HLPNs@TMZ	**	**	**
	HLPNs vs FAM-HLPNs@TMZ	N.S.	N.S.	N.S.
	free TMZ vs LinTT1-HLPNs@TMZ	**	*	*
	free TMZ vs FAM-HLPNs@TMZ	N.S.	N.S.	N.S.
	LinTT1-HLPNs@TMZ vs FAM-HLPNs@TMZ	*	**	N.S.

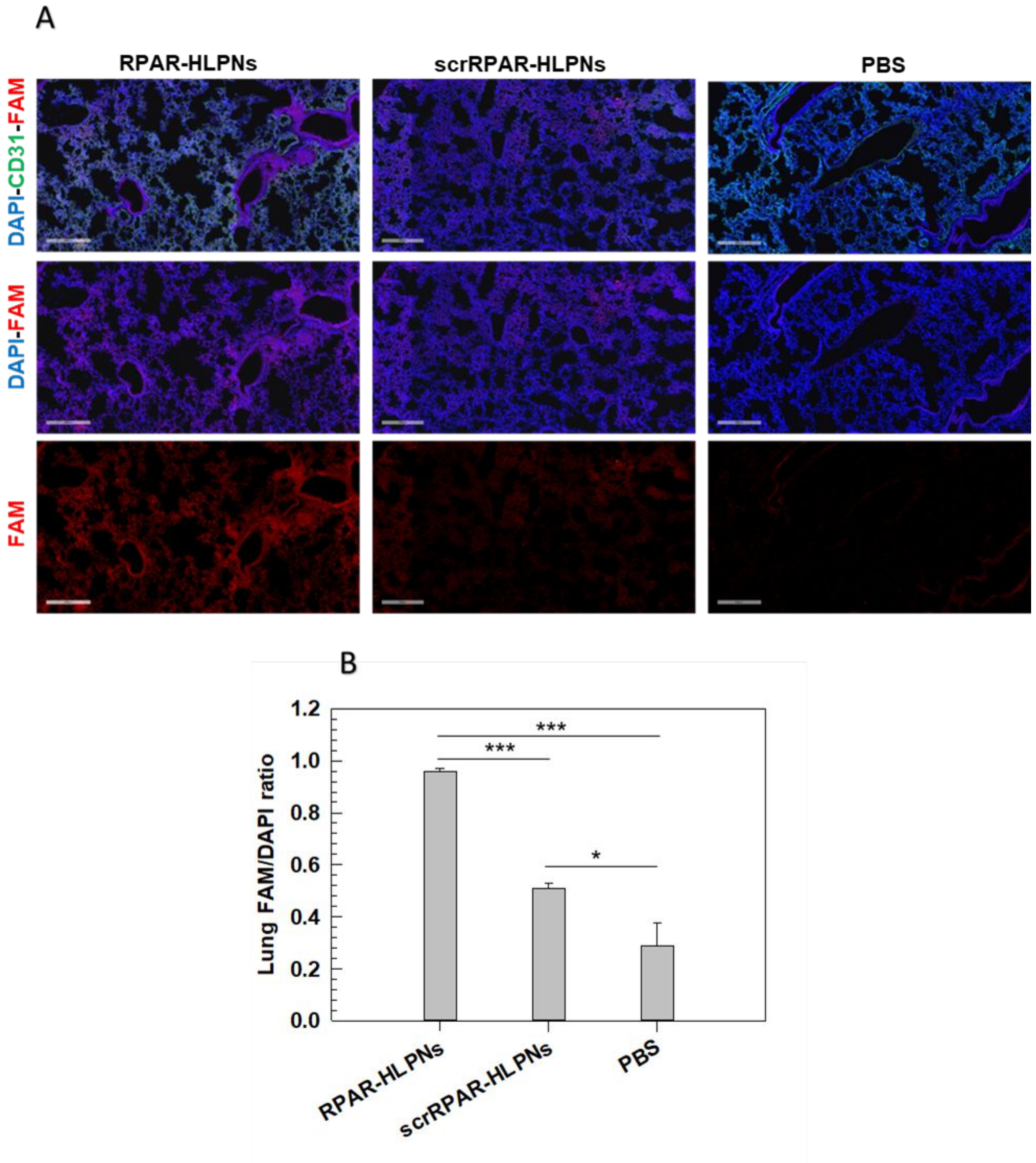

Figure S4. ***In vivo* homing studies of peptide targeted HLNPs to lung tissue**. **(A)** Confocal fluorescence imaging of the lungs injected with RPAR- or scrRPAR-HLPNs. Tissues were extracted 1-hour post-injection of HLPNs, sectioned, and immune-stained for FAM, CD31 and the nuclei were stained with DAPI. The red signal represents RPAR- or scrRPAR- HLPNs, the green signal represents blood vessels (CD31), and blue signal represents nuclei (DAPI). **(B)** RPAR- or scrRPAR- HLPNs signal quantification in the lungs 1-hour post-injection, n = 6 (different areas of the same section, n = 2 mice per group). *$p < 0.05$. **$p < 0.01$. ***$p < 0.001$ [one-way analysis of variance (ANOVA)].

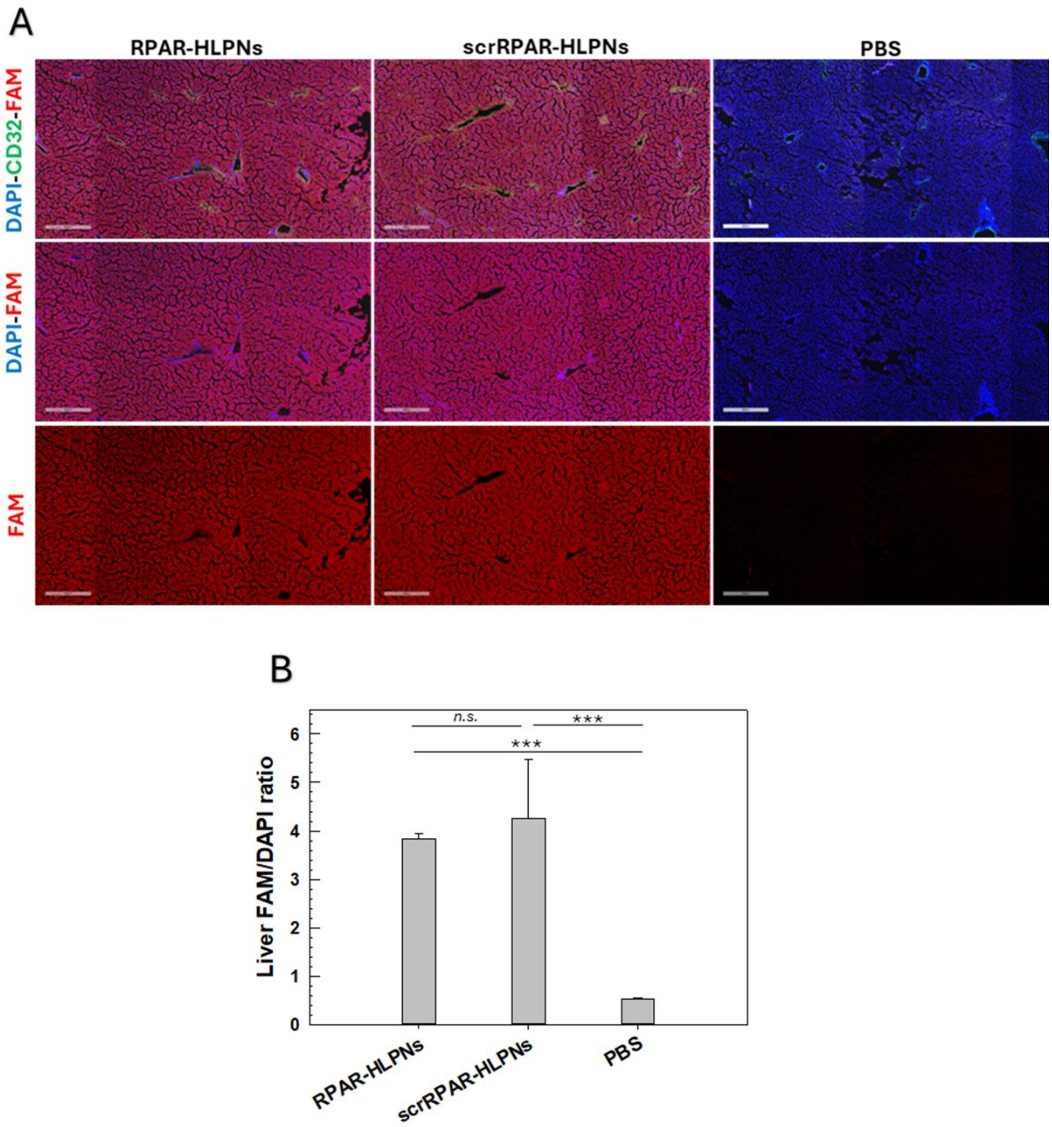

Figure S5. *In vivo* biodistribution of untargeted HLPNs. **(A)** Confocal fluorescence imaging of liver sections collected 1 hour post-injection of RPAR- or scrRPAR-HLPNs in healthy mice. Tissues were sectioned and immuno-stained for FAM (red), CD31 (green, blood vessels), and nuclei stained with DAPI (blue). The red fluorescence signal corresponds to untargeted nanoparticle hepatic accumulation. Scale bar: 400 µm. **(B)** Quantification of RPAR- or scrRPAR-HLPNs signal in the liver, n = 6 (different areas of the same section, n = 2 mice per group). $^{*}p < 0.05$, $^{**}p < 0.01$, $^{***}p < 0.001$ [one-way analysis of variance (ANOVA)].

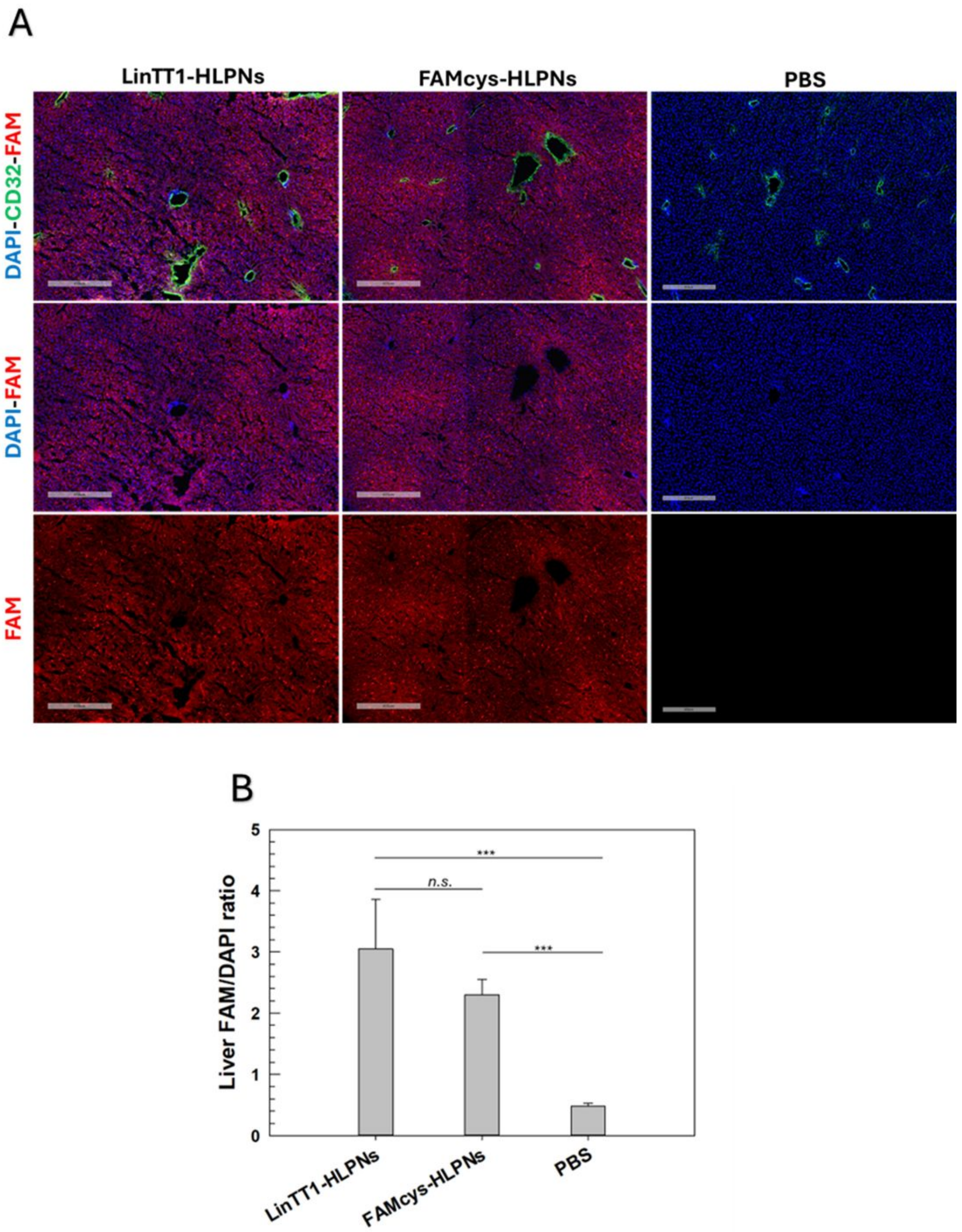

Figure S6 ***In vivo*** **hepatic accumulation of untargeted HLPNs in VEGFko-GBM tumor-bearing mice. (A)** Confocal fluorescence imaging of liver sections collected 3 hours post-injection of LinTT1-HLPNs, FAMcys-HLPNs, or PBS. Tissues were sectioned and immuno-stained for FAM (red), CD31 (green, blood vessels), and nuclei were stained with DAPI (blue). Scale bar: 400 µm. **(B)** Quantification of HLPNs fluorescence signal in liver tissue 3 hours post-injection, n = 6 (different areas of the same section, n = 2 mice per group). *p < 0.05, **p < 0.01, ***p < 0.001 [one-way analysis of variance (ANOVA)].

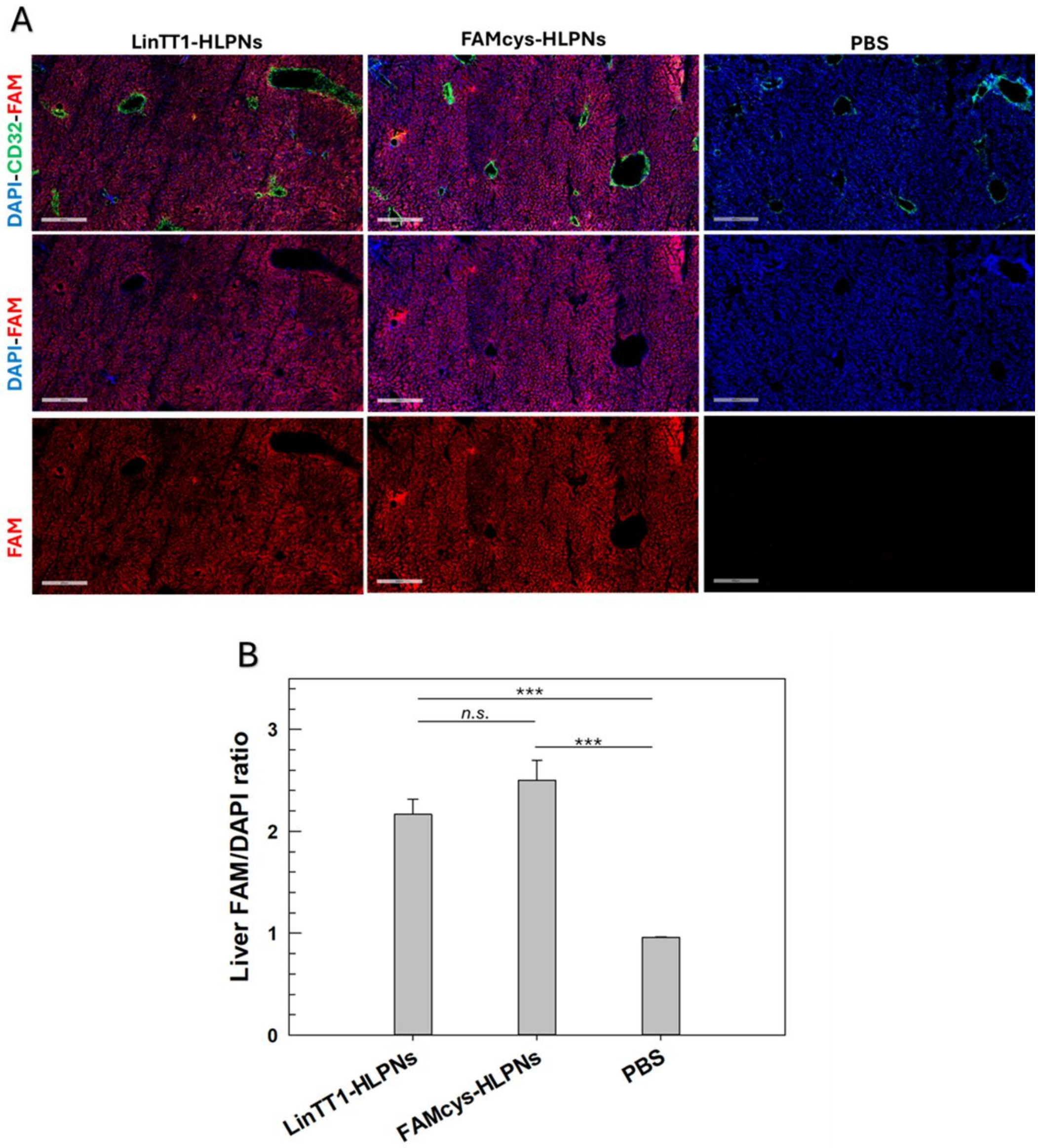

Figure S7 ***In vivo*** **hepatic accumulation of untargeted HLPNs in wtGBM tumor-bearing mice. (A)** Confocal fluorescence imaging of liver sections collected 3 hours post-injection of LinTT1-HLPNs, FAMcys-HLPNs, or PBS. Tissues were sectioned and immuno-stained for FAM (red), CD31 (green, blood vessels), and nuclei were stained with DAPI (blue). Scale bar: 400 µm. **(B)** Quantification of HLPNs fluorescence signal in liver tissue 3 hours post-injection, n = 6 (different areas of the same section, n = 2 mice per group). $*p < 0.05$, $**p < 0.01$, $***p < 0.001$ [one-way analysis of variance (ANOVA)].
